# Molecular Evolution of Tooth-Related Genes Provides New Insights into Dietary Adaptations of Mammals

**DOI:** 10.1007/s00239-021-10017-1

**Published:** 2021-07-21

**Authors:** Yuan Mu, Ran Tian, Linlin Xiao, Di Sun, Zepeng Zhang, Shixia Xu, Guang Yang

**Affiliations:** Jiangsu Key Laboratory for Biodiversity and Biotechnology, College of Life Sciences, Nanjing Normal University, Nanjing, 210023 China

**Keywords:** Feeding habits, Positive selection, Parallel evolution, Association

## Abstract

Mammals have evolved different tooth phenotypes that are hypothesized to be associated with feeding habits. However, the genetic basis for the linkage has not been well explored. In this study, we investigated 13 tooth-related genes, including seven enamel-related genes (*AMELX*, *AMBN*, *ENAM*, *AMTN*, *ODAM*, *KLK4* and *MMP20*) and six dentin-related genes (*DSPP*, *COL1A1*, *DMP1*, *IBSP*, *MEPE* and *SPP1*), from 63 mammals to determine their evolutionary history. Our results showed that different evolutionary histories have evolved among divergent feeding habits in mammals. There was stronger positive selection for eight genes (*ENAM*, *AMTN*, *ODAM*, *KLK4*, *DSPP*, *DMP1*, *COL1A1*, *MEPE*) in herbivore lineages. In addition, *AMELX*, *AMBN*, *ENAM*, *AMTN*, *MMP20* and *COL1A1* underwent accelerated evolution in herbivores. While relatively strong positive selection was detected in *IBSP*, *SPP1*, and *DSPP*, accelerated evolution was only detected for *MEPE* and *SPP1* genes among the carnivorous lineages. We found positive selection on *AMBN* and *ENAM* genes for omnivorous primates in the catarrhini clade. Interestingly, a significantly positive association between the evolutionary rate of *ENAM*, *ODAM*, *KLK4*, *MMP20* and the average enamel thickness was found in primates. Additionally, we found molecular convergence in some amino acid sites of tooth-related genes among the lineages whose feeding habit are similar. The positive selection of related genes might promote the formation and bio-mineralization of tooth enamel and dentin, which would make the tooth structure stronger. Our results revealed that mammalian tooth-related genes have experienced variable evolutionary histories, which provide some new insights into the molecular basis of dietary adaptation in mammals.

**Supplementary Information:**

The online version contains supplementary material available at 10.1007/s00239-021-10017-1.

## Background

Teeth are vital organs responsible for survival and diversity in vertebrates due to their function in cutting, grinding, and/or crushing food as well as their use in attacking and defense (Bergqvist 2003). Mammalian lineages have evolved complex and variable dentitions to adapt to the broad array of diets and environments (Stokstad 2001). Thus, there is a good correlation between feeding habits (herbivorous, omnivorous and carnivorous), patterns of tooth formation (e.g., cardiform, villiform, incisor, canine, molariform) and their complex and highly variable dentition in mammals (Walker et al. 1978; Koussoulakou et al. 2009; Ungar 2010). Additionally, mammalian teeth are widely used in the studies of ecology, paleontology, functional morphology, systematics, and adaptation of feedings in different lineages, such as herbivores, carnivores and omnivores.

For herbivores, such as rodents and artiodactyls, their molars have evolved into hypsodont, lophodont and selenodont shapes, which are beneficial for fiber-mastication. By contrast, the most typical teeth of carnivores are blade-like and work together to provide a scissoring action for hunting, catching and killing prey as well as shearing flesh. Most omnivorous molars are bunodont; hominids have thicker enamel, and cercopithecids have bilophodonts with high crowns (Line 2010; Ungar 2010). Different lineages that feed on similar diets may have evolved convergent tooth shapes, structures and developmental types, such as continuous growth found in most herbivorous species (Simon 2005; Ungar 2010). However, despite their importance for animal survival, teeth have been lost independently in multiple lineages, such as baleen whales, pangolins and adult Monotremes. In addition, some lineages have lost their enamel, such as pygmy and dwarf sperm whales, sloths, armadillos and aardvarks, independently. It was probably due to highly specialized feeding habits and strategies (Werth 2000; Tiphaine et al. 2009). For example, baleen whales lost their teeth and evolved baleen to filter food (Uhen 2010). In addition, teeth development is related to mammalian evolution history and phylogeny. This “phylogenetic baggage” may complicate the functional adaptation of teeth (Ungar 2010).

Teeth are typically composed of two main components, enamel and dentin. The microstructure changes of enamel and dentin can also reflect mammalian feeding adaptations. For example, enamel rods, the basic unit of enamel, are rectangular in sheep and rabbits (herbivorous), columniform in dogs (carnivorous), and intermediate in humans (omnivorous) (Bradford 1967; Shimoyama 1967; Hua 2015).

Mammalian dentition develops through a series of well-defined morphological stages (bud stage, cap stage and bell stage) (Zhang et al. 2005a, b), which are regulated and controlled by a series of genes (Thesleff 2006; Bei 2009). So far, more than 300 genes are reported to be associated with tooth development. Previous researches have confirmed some genes play key roles in enamel matrix formation, organization, and mineralization, such as, amelogenin X-linked (*AMELX*), ameloblastin (*AMBN*), enamelin (*ENAM*), amelotin (*AMTN*) (Termine et al. 1980; Sasaki and Shimokawa 1995; Girondot and Sire 1998; Lu et al. 2008; Al-Hashimi et al. 2009; Neves et al. 2012; Delsuc et al. 2015; Gasse et al. 2015; Fouillen et al. 2017). In addition, some crucial metalloproteases genes take part in hydrolysis and biomineralization of enamel proteins, including matrix metalloproteinase 20 (*MMP20*) and kallikrein 4 (*KLK4*) (Lu et al. 2008). These genes are termed as “enamel-related genes”. On the other hand, some “dentin-related genes”, dentin sialophosphoprotein (*DSPP*) and collagen type I (*COL1*) are major genes for dentin key component, other genes are involved in dentinogenesis (Kawasaki et al. 2007; Yamakoshi 2008), such as dentin matrix acidic phosphoprotein 1 (*DMP1*), integrin binding sialoprotein (*IBSP*), matrix extracellular phosphoglycoprotein (*MEPE*), and secreted phosphoprotein 1 (*SPP1*). Based on previous studies, *AMELX*, *AMBN*, *ENAM*, *AMTN*, *ACPT*, *ODAM*, *KLK4*, *MMP20* and *DSPP* have been inactivated in edentulous and enamel-less mammals, or process of severe amelogenesis imperfecta (AI), or abnormal dentin production and mineralization. These genes might be due to expression evolution of expression-profiles or different mutations, such as premature stop codons, frameshift indels, splice site mutations, etc. (Gibson et al. 2001; Xiao et al. 2001; Crawford et al. 2007; Mcknight and Fisher 2009; Meredith et al. 2009, 2011; Goldberg et al. 2011; Gasse et al. 2012; Delsuc et al. 2015; Machado et al. 2016; Smith et al. 2017; Springer et al. 2019; Mu et al. 2021).

Phenotypes are regulated by both genotype and environment. For teeth, there is a significant correlation between tooth shapes and feeding habits, the linkage between feeding habits and gene evolution is poorly studied. Some gene’s evolution is confirmed that related to dietary adaptation (Kelley and Swanson 2008). However, the molecular evolutionary basis for teeth formation corresponding to feeding adaptations (herbivore, omnivore, carnivore) has not been well explored thus far. In the present study, we investigated the evolution of 13 candidate genes, including seven enamel-related genes (*AMELX*, *AMBN*, *ENAM*, *AMTN*, *ODAM*, *KLK4* and *MMP20*) and six dentin-related genes (*DSPP*, *COL1A1*, *DMP1*, *IBSP*, *MEPE* and *SPP1*) (detailed information on each gene is presented in supplementary Table S1) from 63 mammalian species with different feeding habitats. Our goal was to determine the evolutionary histories of these tooth-related genes and to provide novel insights into the genetic mechanisms of tooth development in mammals with different feeding habits.

## Results

### Convergent/Parallel Sites Among Different Dietary Mammal Lineages

**Fig. 1:**
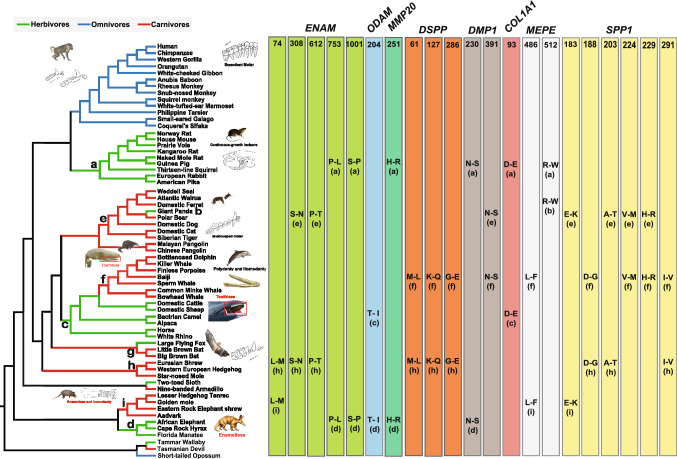
Parallel amino acid changes on the phylogenetic tree. Amino acid positions are listed on the top of colored bars (numbers), and parallel changes at each position were listed in the right part of colored bars corresponding to genes marked in different colors. Different colorful shades on phylogenetic tree indicate different feedings, green is herbivorous, blue is omnivorous, red is carnivorous. Pictures shown in right side exhibit the characters of each dietary mammalian teeth. (Images are obtained from Hillson 2005; WiKi: https://en.wikipedia.org/wiki/Main_Page; AWD website: http://animaldiversity.org/; Baleen whale: https://www.salariya.com/web_books/whaling/intro/baleen.html; Pangolin skull: http://pierce.wesleyancollege.edu/faculty/brhoades/WOC/mammals/pangolin.html)

We identified convergent/parallel amino acid changes in 13 tooth-related genes which suggests molecular convergent evolution based on feeding habit. A total of 21 parallel nonsynonymous amino acid substitutions were identified. Of these sites, seven parallel sites were found among herbivorous lineages at six genes (*ENAM*, *ODAM*, *MMP20*, *DMP1*, *COL1A1*, *MEPE*), and 14 parallel sites were found among carnivorous lineages at five genes (*ENAM*, *DSPP*, *DMP1*, *MEPE*, *SPP1*) (Fig. 1 and Table 1). These parallel substitutions deviated significantly from the random expectation at a significance level of *P* < 0.05.

**Table 1:** Statistical tests for parallel nonsynonymous amino acid substitutions among different dietary lineages

Genes	Branch pair*	Parallel substitution	Observed number	Expected number	*P-*value
*ENAM*	a vs. d	753 P–L	5	0	0
		1001 S–P			
	e vs. h	308 S–N			
		621 P–T			
	h vs. i	74 L–M			
*ODAM*	c vs. d	204 T–I	1	0	0
*MMP20*	a vs. d	251 H–R	1	0	0
*DSPP*	f vs. h	61 M–L	3	0	0
		127 K–Q			
		286 G–E			
*DMP1*	a vs. d	230 N–S	2	0	0
	e vs. f	391 N–S			
*COL1A1*	a vs. c	93 D–E	1	0	0
*MEPE*	a vs. b	512 R–W	2	0	0
	f vs. i	486 L–F			
*SPP1*	e vs. i	183 E–K	6	0	0
	e vs. h	203 A–T			
	e vs. f	224 V–M			
		229 H–R			
	f vs. h	188 D–G			
		291 I–V			

The parallel substitution amino acid sites are detected by PAML, and the *P*-value was calculated by CONVERG 2

*The branch pair letters correspond to Fig. 1

### Detection of Selective Pressure

**Fig. 2:**
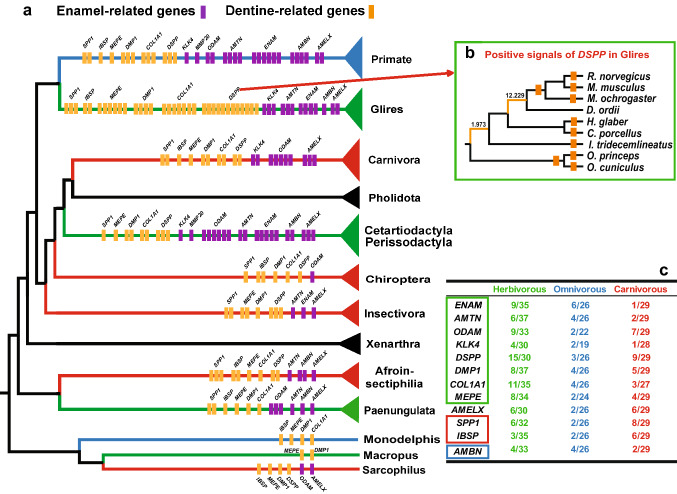
**a** Summary of positive selection of 13 tooth-related genes base on free-ratio model and branch-site model. Every small bar on each clade of tree means one positive selected branch was detected in corresponding clade (small purple bar stood for enamel-related genes, small orange bar stood for dentin-related genes). **b** Distribution of positive signals of DSPP in Glires. **c** The proportion of positively selected branches of each gene in different dietary lineages (Denominator is the number of branches for each different dietary lineage respectively, numerator is the number of positively selected branches for different dietary lineage respectively). Green, blue and red colour showed above represent herbivorous, omnivorous and carnivourous feeding habits, respectively

The results showed that the free-ratio model (Model K) fit the data significantly better than the one-ratio model (Model A) for 13 tooth-related genes (supplementary Table S2), suggesting divergent selective pressures occurred on different mammalian branches. In addition, ω values greater than one were identified in several specific lineages for 13 tooth-related genes (supplementary Figure S1a-l). Simultaneously, the branch-site model also showed that the alternative hypothesis (Ma) was significantly better than the null hypothesis (Ma0) for some branches among 13 tooth-related genes (supplementary Table S3). When we integrated the results from the free-ratio model and branch-site model, a relatively stronger selection intensity (more branches were under positive selection) was detected in herbivorous lineages at eight genes (*ENAM*: 9/35, *AMTN*: 6/37, *ODAM*: 9/33, *KLK4*: 4/30, *DSPP*: 15/30, *DMP1*:9/37, *COL1A1*: 11/35 and *MEPE*: 8/34) than in carnivorous (*ENAM*: 1/29, *AMTN*: 2/29, *ODAM*: 7/29, *KLK4*: 1/28, *DSPP*: 9/29, *DMP1*:5/29, *COL1A1*: 3/27 and *MEPE*: 4/29) and omnivorous (*ENAM*: 6/26, *AMTN*: 4/26, *ODAM*: 2/22, *KLK4*: 2/19, *DSPP*: 3/26, *DMP1*:4/26, *COL1A1*: 4/26 and *MEPE*: 2/24) lineages (Fig. 2a, c). On the other hand, we found a stronger positive selection in carnivorous linages at *IBSP* (6/29) and *SPP1* (8/29) (Fig. 2a, c). Interestingly, a relatively strong positive selection was also detected in the catarrhine clade of omnivorous lineages at *AMBN* (4/26) and *ENAM* (6/26). Additionally, positive selection was detected along some cetacean branches at *AMELX*, *AMBN*, *AMTN*, *MMP20*, *COL1A1*, *DMP1*, *MEPE*, *IBSP* and *SPP1* (supplementary Figure S2).

**Fig. 3:**
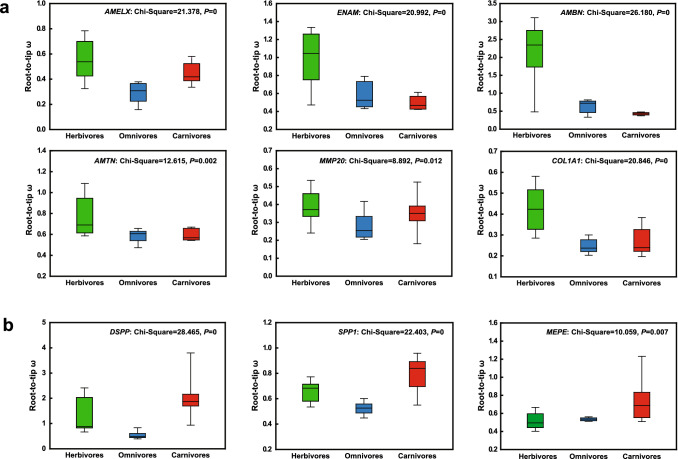
Comparison of evolutionary rates of related genes among three feeding types within mammalian lineages. **a** The evolutionary rates of herbivores are significantly higher than carnivores and omnivores, and **b** the evolutionary rates of carnivores are significantly higher than herbivores and omnivores

The root-to-tip ω ratios of 13 tooth-related genes were calculated to compare the evolutionary rate of tooth-related genes for three feeding types within mammal lineages. It showed that the root-to-tip ω of five enamel-related genes (*AMELX*, *AMBN*, *ENAM*, *AMTN*, *MMP20*) and one dentin-related gene (*COL1A1*) were highest in herbivores, whereas the three dentin-related genes (*DSPP*, *MEPE* and *SPP1*) showed highest *ω* values in carnivores (Fig. 3a, b). The Kruskal–Wallis test further showed significant differences in evolutionary rates in over nine genes among three dietary mammalian lineages (supplementary Table S4), suggesting that some tooth-related genes went through accelerated evolution in specific feeding habitat lineages.

### Association Between Genes Evolution and Enamel Thickness in Primates

**Fig. 4:**
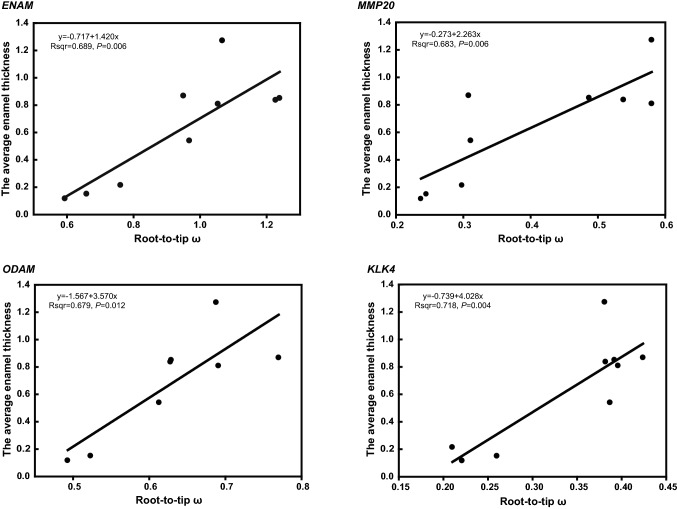
Associations between root-to-tip ω and average enamel thickness among primates. The *ENAM*, *ODAM*, *MMP20*, *KLK4* play key roles in amelogenesis. *ENAM* encodes the largest protein in the enamel matrix of developing teeth. *ODAM* plays a role in odontogenesis and development of enamel matrix. *MMP20* and *KLK4* take part in the hydrolysis of enamel protein matrix and enamel biomineralization. (The relative information is from GeneCard database: https://www.genecards.org/)

To explore the relationship between the evolutionary rate of tooth-related genes and the tooth phenotype, we implemented PGLS to estimate the association between the root-to-tip ω of enamel-related genes and the average enamel thickness in primates (dataset III, supplementary Figure S3). We found that PGLS analysis generated a *λ* value of 0, indicating no phylogenetic signal in our analysis (supplementary Table S5). Thus, only OLS (Ordinary Least Squares) was carried out, and the results showed a significant positive regression between the root-to-tip *ω* and average enamel thickness at *ENAM*, *ODAM*, *MMP20*, and *KLK4* (Fig. 4), whereas no significant association was found in *AMELX*, *AMBN* and *AMTN*.

### Pseudogenization in Toothless and Enamel-Less Mammals

In this study, 13 genes were used. Of these genes, eight genes (*AMELX*, *AMBN*, *ENAM*, *AMTN*, *ODAM*, *MMP20*, *KLK4*, *DSPP*) were detected to be lost or pseudogenes among the enamel-less and toothless mammals. They are mainly due to premature stop codons, frameshift indels, splice site mutations etc. (supplementary Figure S4), most of which was consistent with previous studies. In addition, premature stop codons were also identified in some toothed species with enamel, such as *AMELX* and *KLK4* in the Yangtze finless porpoise (*Neophocaena asiaeorientalis*), *MEPE* in the galago (*Otolemur garnettii*) and naked mole rat (*Heterocephalus glaber*). The tooth-related genes in other species were found to be intact.

We further detected whether relaxation of selective pressure occurred in the pseudogenized edentulous/enamel-less lineages. The results showed the selective pressure in some lineages with pseudogenes have already been relaxed (Table 2), as well as the functional constraints on *AMBN*, *ENAM*, *MMP20* and *DSPP* were completely removed in some enamel-less and/or toothless lineages (Table 2). However, no significant selective relaxation was detected in toothless / enamel-less lineages at *AMELX*, *ODAM* and *KLK4*.

**Table 2:** Likelihood and omega values estimated under two ratio branch model of selective pressures on tooth-related genes

Models and special branches	*ω*	− ln L	np	Models comparison	2Δ (ln L)	*P*-value
*AMBN*						
A. All branches have one *ω*	0.459	22,143.52	124			
B. All branches have one *ω* = 1	1	22,429.33	123	B vs A	571.622	0
C. The terminal branch of *Orycteropus afer afer* with pseudogenized *AMBN* has *ω*_2_, others have *ω*_1_	*ω*_1_ = 0.454 *ω*_2_ = 0.894	22,140.4	125	A vs C	6.224	0.013
D. The terminal branch of *Orycteropus afer afer* with pseudogenized *AMBN* has *ω*_2_ = 1, others have *ω*_1_	*ω*_1_ = 0.454 *ω*_2_ = 1	22,140.48	124	D vs C	0.156	0.693
E. The ancestral branch of Pholidota has *ω*_2_, others have *ω*_1_	*ω*_1_ = 0.450 *ω*_2_ = 1.009	22,136.21	125	A vs E	14.618	< 0.001
F. The ancestral branch of Pholidota has *ω*_2_ = 1, others have *ω*_1_	*ω*_1_ = 0.450 *ω*_2_ = 1	22,136.21	124	F vs E	0.002	0.964
*ENAM*						
A. All branches have one *ω*	0.472	64,566.98	128			
B. All branches have one *ω* = 1	1	65,277.19	127	B vs A	1420.43	0
C. The terminal branch of *Manis javanica* with pseudogenized *ENAM* has *ω*_2_, others have *ω*_1_	*ω*_1_ = 0.470 *ω*_2_ = 1.034	64,563.42	129	A vs C	7.112	0.008
D. The terminal branch of *Manis javanica* with pseudogenized *ENAM* has *ω*_2_ = 1, others have *ω*_1_	*ω*_1_ = 0.470 *ω*_2_ = 1	64,563.43	128	D vs C	0.01	0.92
E. The ancestral branch of Pholidota has *ω*_2_, others have *ω*_1_	*ω*_1_ = 0.468 *ω*_2_ = 0.806	64,561.14	129	A vs E	11.67	< 0.001
F. The ancestral branch of Pholidota has *ω*_2_ = 1, others have *ω*_1_	*ω*_1_ = 0.468 *ω*_2_ = 1	64,561.98	128	F vs E	1.674	0.196
G.The terminal branch of *Choloepus hoffmanni* with pseudogenized *ENAM* has *ω*_2_, others have *ω*_1_	*ω*_1_ = 0.469 *ω*_2_ = 0.914	64,562.17	129	A vs G	9.61	0.002
H. The terminal branch of *Choloepus hoffmanni* with pseudogenized *ENAM* has *ω*_2_ = 1, others have *ω*_1_	*ω*_1_ = 0.469 *ω*_2_ = 1	64,562.25	128	H vs G	0.154	0.695
I. The terminal branch of *Dasypus novemcinctus*with pseudogenized *ENAM* has *ω*_2_, others have *ω*_1_	*ω*_1_ = 0.468 *ω*_2_ = 0.743	64,562.4	129	A vs I	9.162	0.002
J. The terminal branch of *Dasypus novemcinctus* with pseudogenized *ENAM* has *ω*_2_ = 1, others have *ω*_1_	*ω*_1_ = 0.467 *ω*_2_ = 1	64,564.07	128	J vs I	3.346	0.067
*MMP20*						
A. All branches have one *ω*	0.18	20,392.24	128			
B. All branches have one *ω* = 1	1	21,668.04	127	B vs A	2551.59	0
C. The terminal branch of *Manis javanica* with pseudogenized *MMP20* has *ω*_2_, others have *ω*_1_	*ω*_1_ = 0.179 *ω*_2_ = 0.864	20,388.99	129	A vs C	6.502	0.011
D. The terminal branch of *Manis javanica* with pseudogenized *MMP20* has *ω*_2_ = 1, others have ω_1_	*ω*_1_ = 0.179 *ω*_2_ = 1	20,389.01	128	D vs C	0.043	0.836
E. The ancestral branch of Pholidota has *ω*_2_, others have *ω*_1_	*ω*_1_ = 0.174 *ω*_2_ = 0.966	20,372.19	129	A vs E	40.104	< 0.001
F. The ancestral branch of Pholidota has *ω*_2_ = 1, others have *ω*_1_	*ω*_1_ = 0.174 *ω*_2_ = 1	20,372.2	128	F vs E	0.012	0.913
*DSPP*						
A. All branches have one *ω*	0.385	58,713.76	114			
B. All branches have one *ω* = 1	1	59,730.58	113	B vs A	2033.64	0
C. The terminal branch of *Dasypus novemcinctus* with pseudogenized *DSPP* has *ω*_2_, others have *ω*_1_	*ω*_1_ = 0.380 *ω*_2_ = 0.771	58,702.61	115	A vs C	22.298	< 0.001
D. The terminal branch of *Dasypus novemcinctus* with pseudogenized *DSPP* has *ω*_2_ = 1, others have *ω*_1_	*ω*_1_ = 0.380 *ω*_2_ = 1	58,704.01	114	D vs C	2.786	0.095

## Discussion

### Convergence Between Unrelated Mammals with the Same Dietary Type

Some deeply divergent mammalian lineages have evolved similar feeding habits during mammalian evolution. For example, glires, perissodactyla, cetartiodactyla and paenungulata have convergently evolved to feed on plants (Fig. 1). To adapt the similar feeding habits, these deeply diverged herbivorous lineages (green branches in the Fig. 1) have evolved convergent tooth phenotype, such as continuous growth, high crown molars, and similar microstructures, etc. that contribute to resistance abrasion from fibrous food (Williams and Kay 2001; Evans and Sanson 2003; Simon 2005; Ungar 2010). Six parallel amino acid nonsynonymous substitutions sites were examined in five genes (*ENAM*, *ODAM*, *MMP20*, *DMP1* and *COL1A1*) among these herbivorous lineages with convergent tooth phenotype.

Similarly, 14 parallel amino acid sites were identified among carnivorous lineages (red branches in Fig. 1). Of these sites, 11 parallel sites were enriched in dentin-related genes (*DSPP*, *DMP1*, *MEPE* and *SPP1*) whereas only three parallel sites examined in the enamel-related gene (*ENAM*). Reasonable explanation of these parallel sites was from the common ancestor. We cannot completely exclude the possibility that these parallel sites were from convergent adaption similar diets. For example, one parallel sites of *ENAM* was identified in the branch h (the ancestral branch of eulipotyphla) and branch i (the ancestral branch of afroinsectiphilia) in Fig. 1. Both groups have evolved similar diet, which mainly feed on insects. To cope with the tough exoskeleton of insects, powerful enamel seems to be particularly important for small insectivores. ENAM is the most important protein for enamel formation, besides, the mineral forms on dentin is not true enamel and easily crumbles in *ENAM*^−/−^ mice (Smith et al. 2009). However, it is difficult to conclusively link phenotypic convergence with some parallel sites identified in these deeply divergent lineages. In future, the function of parallel sites identified in these deeply divergent lineages is needed to clarify.

### Evolutionary Histories of Tooth-Related Genes in Different Dietary Mammals

To offset the abrasion, teeth of herbivorous mammals have evolved specific traits, such as high tooth crown, continuous growth, etc., which is the effective mechanisms to better cope with abrasion. The herbivores need much harder enamel and tooth structure to offset the abrasion. Correspondingly, the selective pressure analyses showed that relatively strong selection intensity was examined in herbivorous lineages in tooth-related genes. Eight out of 13 tooth-related genes (*ENAM*, *AMTN*, *ODAM*, *KLK4*, *DSPP*, *DMP1*, *COL1A1* and *MEPE*) were under the much stronger positive selection. Among these genes, *ENAM*, *AMTN* and *KLK4* are involved in the development, formation and mineralization of enamel, and ENAM protein is an important component of enamel matrix proteins (Sierant and Bartlett 2012; Smith et al. 2017). *ENAM*^−/−^ mice lacked true enamel, also *KLK4*-knockout mice produce quite brittle enamel (Hu et al. 2008; Simmer et al. 2009). Similarly, tooth enamel was structurally deficient in *AMTN*^−/−^ and *AMTN*^±^ mice (Nakayama et al. 2015). Therefore, we speculated that the positive selection of these genes might promote enamel formation and biomineralization to a certain extent, thus making enamel structure more compact.

Compared with carnivorous lineages, stronger positive selection in herbivores may contribute to the formation of better enamel layer to enhance the resistance to food fibers, improve the wear resistance of teeth, and thus increase the service life of teeth. On the other hand, DSPP is the most important non-collagen protein in dentin, which participates in dentin formation and biomineralization, mutation of *DSPP* gene will lead to serious dentin loss (Kim et al. 2005; Yamakoshi 2009). In addition, four dentin-related genes (*DSPP*, *DMP1*, *COL1A1* and *MEPE*) also exhibited much stronger positive selection in herbivores, especially glires (Fig. 2c). This may be related to their dentin, which is exposed and needs to directly resist the abrasion of plant fibers. Beside, accelerated evolution was examined in herbivores lineages in *AMELX*, *AMBN*, *ENAM*, *AMTN* and *MMP20* and *COL1A1*, which well supported previous phenotypic studies that mammals that feed on plants possess higher levels of ‘dental complexity’ than those that feed on animals (Evans et al. 2007; Santana et al. 2011).

Different from the herbivorous lineages, only two dentin-related genes (*IBSP* and *SPP1*) were under the strongly positive selection in carnivorous lineages. In addition, accelerated evolutionary rate was detected in three dentin-related genes (*DSPP*, *SPP1* and *MEPE*) (Fig. 3b), which exhibited obvious differences from herbivorous lineages. In herbivorous lineages, the genes with accelerated evolution mainly focused on enamel-related genes (*AMELX*, *AMTN*, *ENAM*, *MMP20* and *AMBN*) (Fig. 3a). Machado et al. (2016) found some tooth-related genes with higher evolutionary rates associate with phenotypic diversity of teeth in mammals (Machado et al. 2016). The rapid evolution of these genes may provide an important basic for the rapid changes of teeth phenotype.

Of course, tooth phenotype won't be restricted to feeding habits. Other factors, such as chewing pattern, life history, also exert effects on diversity of tooth phenotype. Thus, we will further test whether there is potential relationship between teeth-related gene evolution and these factors in future.

### Evolution of Enamel-Related Genes is Associated with Feeding Habits in Primates

For most omnivorous primates, morphological studies have shown that the enamel layer in anthropoids is much thicker than that in prosimians (Shellis et al. 1998). More food resource is available for mammals with thicker enamel layer, and enamel layer could protect teeth from abrasion, thus increase the working life of teeth (Dumont 1995; Kay 2010). For example, human and chimpanzee have relatively thicker enamel layer to adapt to more food resources (Ungar 1992; Shellis et al. 1998). By contrast, small-eared galago has thin enamel layer so that just feed on fruits and some insects (the detailed information was seen in supplementary Table S6). Thus, enamel thickness could be regarded as a proxy for dietary adaptation to some extent. Statistical association between selection on functional genes and changes in phenotype is an important indication for exploring the genetic basis of adaptive phenotypes (Montgomery et al. 2014). It has been demonstrated that changes in noncoding regions are associated with rapid evolutionary changes in enamel thickness and that they can have a major impact through differentially altering the affinity of transcription factors that regulate tooth development (Horvath et al. 2014). Interestingly, a significantly positive correlation between the evolutionary rate and the average enamel thickness was found in ORF of *ENAM*, *ODAM*, *MMP20* and *KLK4* (Fig. 4). ENAM, ODAM, MMP20 and KLK4 were reported to mediate the formation of enamel and enamel thickening during amelogenesis, enamel will disorder when these gene mutate or be inactive (Crawford et al. 2007; Smith et al. 2017). In addition, stronger intensity of positive selection was identified among catarrhini clade in *AMBN* and *ENAM*. Thus, it was suggested that there is a significant correlation between tooth enamel thickening and evolution of tooth-related genes in primates, which is conducive to tooth enamel formation and thickening. Although average enamel thickness is a crude measure of dietary adaptation, average enamel thickness is relatively valuable data to evaluate the strength of teeth. What these other mechanisms are remains to be determined, and further assessment of the relationships among ameloblasts activity, jaw movements will lead to a better understanding of the evolution of teeth and food choice in primates.

### Inactivation of Tooth-Related Genes in Enamel-Less/Toothless Mammals Probably Duo to Specialized Feeding Habits or Strategies

To adapt to specific diet and environment, some mammalian lineages have lost teeth or enamel independently during evolution (Tiphaine et al. 2009). Anatomical evidence showed that the enamel of extant pygmy / dwarf sperm whale, Cingulata (armadillos), Folivora (sloths) and Tubulidentata (aardvarks) have been degenerated or lost. Accordingly, convergent pseudogenization in the tooth-related genes (*AMELX*, *AMBN*, *ENAM*, *AMTN*, *ODAM*, *MMP20*, *KLK4*, *DSPP*) are essential for enamel and dentin formation.

Due to loss of precise dental occlusion, trends of dental morphology of cetaceans, including mysticeti and odontoceti, have been simplified (Armfield et al. 2013; Peredo et al. 2018). As well as the Hunter–Schreger bands (HSB), which are decussating layers of prisms that increase the strength of enamel, have been lost in the inner enamel layer of living toothed whales(Ishiyama 1987; Loch et al. 2013; Carolina et al. 2015). Springer et al. (2019) found that inactivation of *ODAM* gene in toothed whales was associated with the simplified outer enamel of their teeth (Springer et al. 2019). By contrast, baleen whales have lost their teeth, replaced by cutinized baleen, a novel integumentary structure that was used to bulk-filter feeding. However, the fossil record have showed that tooth phenotype has transformed in stem mysticetes, such as primitive forms that had teeth but not baleen (e.g. *Janjucetus*, *Mammalodon*), intermediate forms that had teeth and baleen (e.g. *Aetiocetus*) and more derived forms with baleen but not teeth (e.g. *Eomysticetus*, *Micromysticetus*) (Sanders and Barnes 2002a, b; Sanders and Barnes 2002a, b; Fitzgerald 2006, 2010; Deméré et al. 2008; Deméré and Berta 2010). Besides, molars are large, multi-cusped, and overlapping and were used for filter feeding in *Coronodon havensteini*, a relative of modern baleen whales but retains teeth, which suggested that filter feeding evolved before baleen (Geisler et al. 2017). Teeth have gradually degenerated probably due to lose of mastication. Interestingly, Meredith et al. (2011) have confirmed that the loss of enamel-capped teeth on the common ancestral branch of crown mysticetes through insertion of a CHR-2 SINE retroposon in exon 2 of *MMP20* in living baleen whales (Meredith et al. 2011). Among extant baleen whales, subsequently, another tooth-related genes (i.e. *AMELX*, *AMBN*, *ENAM*, *ODAM*, *AMTN* and *DSPP*) were examined to be pseudogenized in baleen whales, which is consistent with tooth loss.

Pangolin is another edentulous mammalian lineage, which mainly feeds on ants and termites. Previous study showed that vestigial teeth start to form in embryos but are resorbed prior to birth in Pangolins (Tims 1908). To date, the fossil species are edentulous (Franzen 2005; Meredith et al. 2009). Tooth-related genes have been pseudogenized or lost in this group, such as *ENAM* was inactivating (Meredith et al. 2009), *KLK4* was not attained by BLASTN (lost). *ENAM* became a pseudogene on the stem pangolin, the functional relaxation of *ENAM* gene was reported to occur approximately 54.9–59.4 Mya, which is ~ 8–12 million years older than *Eomanis* (Meredith et al. 2009). Some toothless mammals, like pangolins and baleen whales, also have evolved some compensation mechanism. For example, the long and sticky tongue in pangolins, which is easily catch ants (Tiphaine et al. 2009).

## Conclusions

In this study, we comprehensively investigated 13 tooth-related genes associated with enamel and dentin development in different dietary mammalian lineages. Our results have reminded that the convergent and parallel amino acids substitution also existed between some lineages which have similar feeding habits. Meanwhile, different evolutionary histories have evolved among divergent feeding habits in mammals. Stronger positive selection of both enamel-related and dentin-related genes in herbivore lineages might facilitate form hard enamel layer to enhance resistance to fractures from biting fibrous objects and protect the teeth from abrasion. By contrast, relatively stronger selection was mainly enriched in dentin-related genes in carnivorous lineages. Interestingly, convergent pseudogenization in tooth-related genes (*AMELX*, *AMBN*, *ENAM*, *AMTN*, *ODAM*, *MMP20*, *KLK4*, *DSPP*) in enamel-less and edentulous lineages probably due to specific feeding habits. In summary, mammalian teeth-related genes evolved with different evolutionary histories due to various feeding habits, which provide new insights into the molecular basis of dietary adaptation in mammals.

## Methods

### DNA Sequences Mining and Alignment

The full-length coding sequences (CDS) of 13 tooth-related genes were extracted from the OrthoMaM v10b (OrthoMaM), Ensembl 100 (Ensembl) and NCBI (NCBI) databases (The detailed information seen in supplementary Table S7, and the multiple sequences alignments were upload to FigShare, the download link: https://figshare.com/articles/online_resource/Molecular_Evolution_of_Tooth-related_Genes_Provides_New_Insights_into_Dietary_Adaptations_of_Mammals/14125784). For the orthologous gene sequences of enamel-less / toothless species e.g., baleen whales, pangolins, and aardvark, we searched the candidate genes in public database, and then confirmed these candidate sequences again through blast the genome sequences (supplementary Table S8), which is via the BLASTN algorithms using the well-annotated genes of humans (*Homo sapiens*) and cows (*Bos taurus*) as queries through python scripts that we developed. Each exon sequences were combined manually based on splice sites. The nucleotide and protein sequences were aligned with Muscle in MEGA7.0 (Kumar et al. 2016), and checked by eye.

### Identification of Parallel/Convergent Sites

To determine whether similar histories of molecular evolution occurred in distantly related animals that have adapted to similar feeding habits, we first constructed the ancestral amino acid sequences of each gene using the CODEML program in the PAML package (Yang 2007). We then searched for parallel/convergent amino acid substitutions between the ancestor branches among convergent feeding habits, i.e., herbivorous lineages (branch a–d in Fig. 1) and carnivorous lineages (branch e–i in Fig. 1), respectively. The software CONVERG 2 (Zhang and Kumar 1997) was used to test whether the observed convergent amino acid substitutions in the focal branches were fixed randomly or by natural selection.

### Analysis of the Selective Pressure

Analyses of the selective pressure were performed based on the ratio of non-synonymous (*d*_N_) to synonymous (*d*_S_) substitutions (*d*_N_/*d*_S_ or ω) in the CODEML program incorporated in the PAML 4.7a package (Yang 2007), where *ω* < 1, *ω* = 1 and *ω* > 1 indicate purifying selection, neutral selection and positive selection, respectively. The well-known species tree (Fig. 1) only served as a guide tree in subsequent PAML analyses (Flynn et al. 2005; Blanga-Kanfi et al. 2009; Kuntner et al. 2011; Polina et al. 2011; Zhou et al. 2012). Here, the premature stop codons were considered to be missing data (regarded as NNN or —) to maintain the sequence integrity for *d*_N_/*d*_S_ analyses with CODEML.

To test for possible heterogeneity of ω ratios along independent dietary branches, we used the free-ratio model, which allows each branch to have a separate *d*_N_/*d*_S_ value (regarded as Model K in Table S2). The null model is a very strict model called the one-ratio model (M0, model B, all branches have one estimated *ω*) that allows only a single *ω* ratio for all branches. Prior to this analysis, model A (all branches have one *ω*) was compared with model B (all branches have one *ω* and *ω* = 1). To further improve the results of free-ratio model, we further implemented branch-site tests to estimate positive selection affected by a few sites along a specific branch (Zhang et al. 2005a, b). We compared alternative model (Ma), which assumes four classes of sites, especially, allowing codons under positive selection along foreground lineage with *ω*_2_ > 1, to the null hypothesis (Ma0), in which fixed *ω*_2_ = 1. Each related branch of different feedings (green is for herbivorous, blue is for omnivorous, and red is for carnivorous in species tree, Fig. 1) were labelled as the foreground branches, respectively. When the positively selected sites with a posterior probability (PP) ≥ 0.80, they were regarded as candidates for selection. It means the related branches are also under the positive selection. The likelihood ratio test (LRT) with a *χ*^2^ distribution was used to determine which model was statistically significant from the null model at a significant level of *P* < 0.05. For all the analyses, the nested models were compared using a likelihood ratio test (LRT) with a *χ*^2^ distribution was used to determine which model was statistically significant at a significant level of *P* < 0.05. All analyses were run at least twice to ensure convergence.

To know the evolutionary history of different dietary mammal lineages, the results of these two models (free-ratio model and branch-site model) are used to evaluate the strength of positive selection in herbivorous, carnivorous and omnivorous lineages, respectively. Through counting the number of positively selective branches, the proportion of them is calculated among three typical dietary mammals. Denominator is the number of branches for different dietary lineages respectively, and numerator is the number of positively selected branches for different dietary lineages respectively.

To estimate the evolutionary rate of each gene among the different dietary lineages, the root-to-tip ω values were calculated in the CODEML program (Yang 2007). Root-to-tip *ω* is more inclusive of the evolutionary history of a locus (Wolf et al. 2009). It is a mean value that includes ω ratios from an ancestral-root to terminal-tip in a phylogenic clade, which was computed in the two-ratio model (S*d_S_ = 0 and N*d_N_ = 0 were not considered). And, it has been regarded to be a signal of evolutionary trajectory for species (Montgomery et al. 2011). Besides, the root-to-tip *ω* is regarded to be more suitable for regression analysis against phenotypic data from extant species (Montgomery et al. 2011; Xu et al. 2017). The statistical comparison of the evolutionary rates among three different dietary lineages was applied in IBM SPSS Statistics 22 (IBM 2013).

Teeth in odontocetes are used to grab prey due to the loss of precise mastication and some species lost their teeth and/or enamel, in which they may be not subjected to the normal pressures like the rest of mammals. Thus, the evolution of tooth-related genes in these special lineages may not reflect the entire evolutionary trajectories. Therefore, the evolutionary rates and positive selection signals were not considered when we carried out statistical treatments of these special lineages.

To evaluate the selective pressure on tooth-related pseudogenes in enamel-less/toothless lineages and related branches, the *d*_N_/*d*_S_ ratios were also calculated by using two-ratio model (Table 2). Based on the assumption that all branches had a single *ω* value, purifying selection was seen across the tree for these genes according to comparison between model A and model B. To ensure whether the functional constraint was relaxed, further comparison between model A and model C, E, G or I, in which pseudogenized branches had a *ω*_2_, while other branches had a ω_1_. To further evaluate whether selective pressure was completely removed, we performed comparisons between model (C, E, G or I) and model (D, F, H or J) which had a fixed *ω*_2_ = 1 in pseudogenized branches (Table 2).

### Association Analysis Between the Root-to-Tip *ω* and Tooth Phenotype

To explore potential relationships between gene evolution and tooth phenotypes (relative enamel thickness of lower M_1_, obtained from Shellis et al. 1998), we examined the association in the primates dataset (dataset III, supplementary Figure S3) using the method described by Montgomery et al. (2011). The average enamel thickness was calculated as A_E_/L_EDJ_. Here, A_E_ was the area of the enamel, L_EDJ_ was the length of the enamel–dentine junction. The two-ratio model was used again to calculate the average *d*_N_/*d*_S_ ratios which is from the ancestral-root to terminal-tip (root-to-tip *ω*). The evolutionary rates could vary when the branch length was included as a covariate, so we obtained the divergent time of each species as the branch length from the TIMETREE website (TIMETREE). Then phylogenetic generalized least squares (PGLS) analysis was carried out in the R program (Orme et al. 2012) to analyze the relationship between the root-to-tip *ω* and tooth enamel thickness. This method included a parameter estimated by the maximum likelihood method, i.e., lambda (*λ*), which was used to estimate the quantitative measures of the phylogenetic signal, which ranged from zero (no phylogenetic signal) to one (significant phylogenetic signal).

## Supplementary Information

Below is the link to the electronic supplementary material.

Supplementary file1 (XLSX 69 kb)

Supplementary file2—Figure S1 Distribution of positive selection of each gene among three feeding mammals. Dashed line in species tree means there’s no corresponding species and data used in analyses (PDF 1563 kb)

Supplementary file3—Figure S2 Distribution of positive selection on cetacean phylogeny. (images are obtained from WiKi website: https://en.wikipedia.org/) (PDF 3933 kb)

Supplementary file4—Figure S3 The primate phylogeny and dataset used for association analysis between root-to-tip ω and average enamel thickness.(Images are derived from WiKi website: https://en.wikipedia.org/wiki/Primate; AWD website: http://animaldiversity.org/, respectively) (PDF 6730 kb)

Supplementary file5—Figure S4 The overview about the information of inactive mutation in some tooth-related genes (PDF 1242 kb)

## Data Availability

We have uploaded the basic data to the FigShare database. The URL link is: https://figshare.com/articles/online_resource/Molecular_Evolution_of_Tooth-related_Genes_Provides_New_Insights_into_Dietary_Adaptations_of_Mammals/14125784. The data and results generated and analyzed during this study are included in this article and its additional files, including nine tables and 12 figures. Enamel thickness of first lower molar (M1) were obtained from Shellis et al. (1998).

## Abbreviations

AMEL: Amelogenin
AMBN: Ameloblastin
ENAM: Enamelin
AMTN: Amelotin
ODAM: Odontogenic ameloblast-associated
KLK4: Kallikrein 4
MMP20: Matrix metalloproteinase 20
DSPP: Dentin sialophosphoprotein
COL1A1: Collagen type I alpha 1
DMP1: Dentin matrix acidic phosphoprotein 1
MEPE: Matrix extracellular phosphoglycoprotein
IBSP: Integrin-binding sialoprotein
SPP1: Secreted phosphoprotein 1
EMP: Enamel matrix protein
HA: Hydroxyapatite
PAML: Phylogenetic analysis by maximum likelihood
PP: Posterior probability
BS: Bootstrap
M1: First lower molar
PGLS: Phylogenetic generalized least squares
Mya: Million years ago
