# Supplementary figures and images for: Molecular Evolution of Tooth-Related Genes Provides New Insights into Dietary Adaptations of Mammals
Source: J Mol Evol. 2021 Jul 21;89(7):458–71. doi: 10.1007/s00239-021-10017-1 (PMC8318974; doi:10.1007/s00239-021-10017-1)

a

**AMELX**

Free ratio —

Branch-site ■

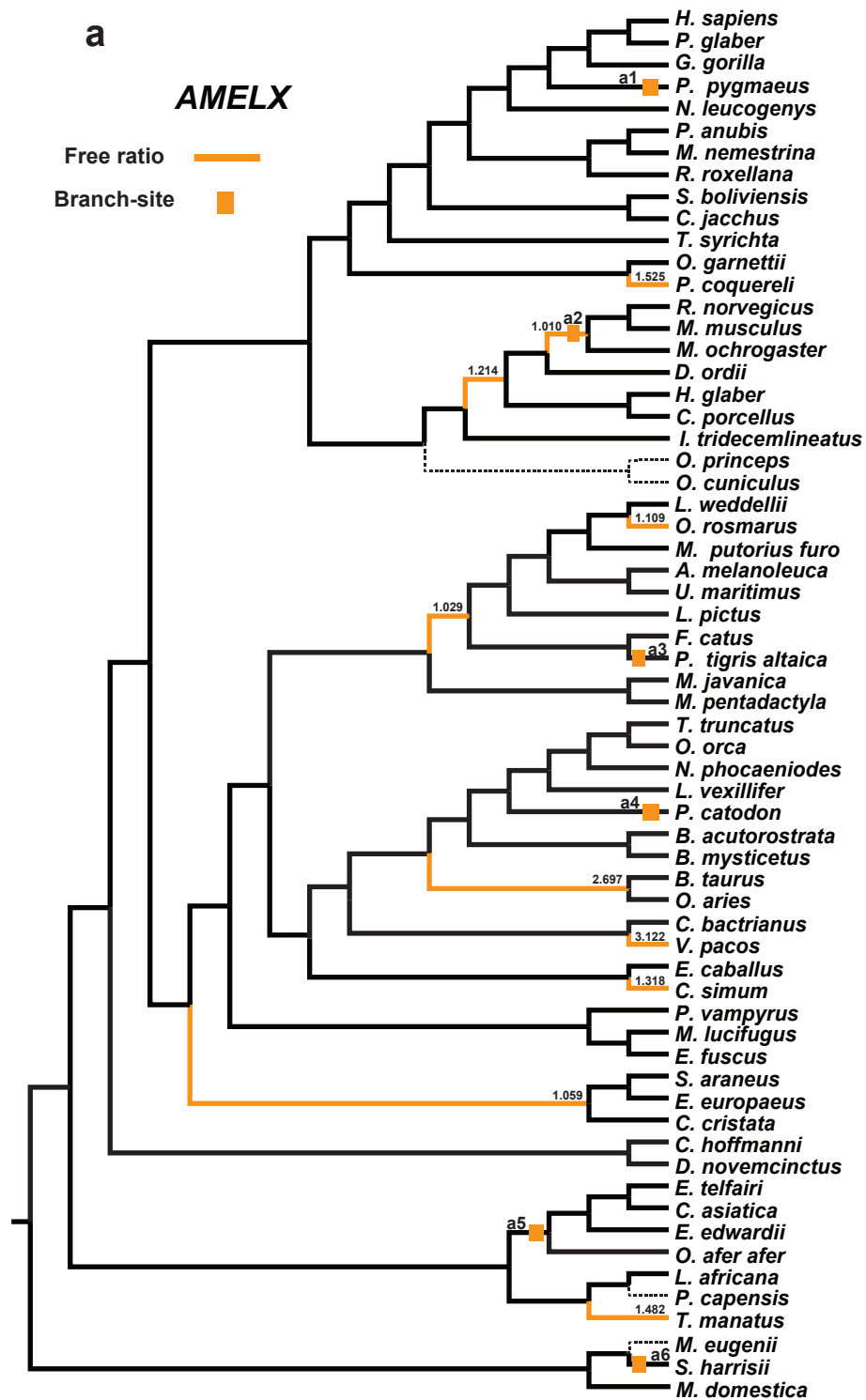

b

**ENAM**

Free ratio —

Branch-site ■

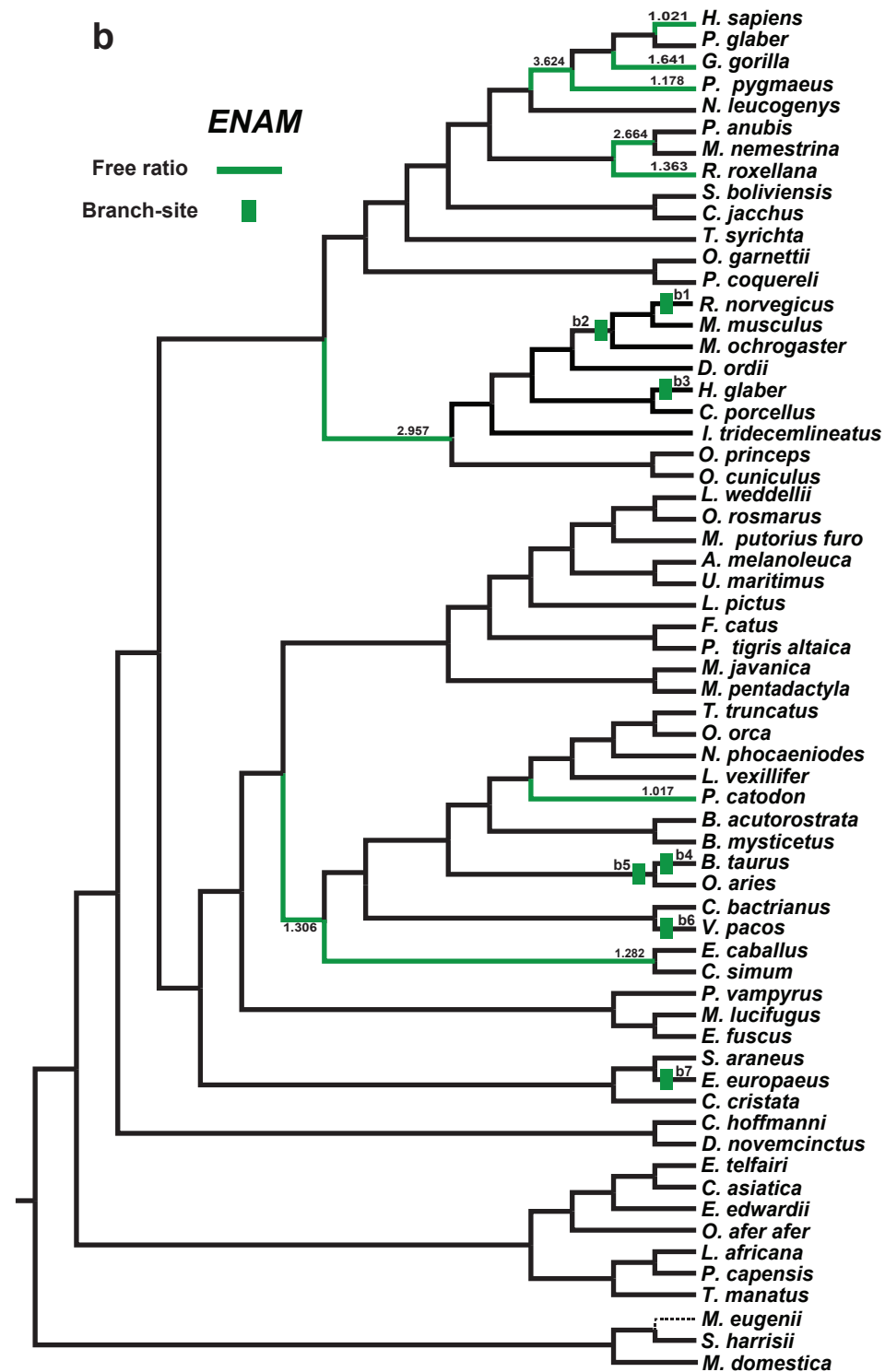

c

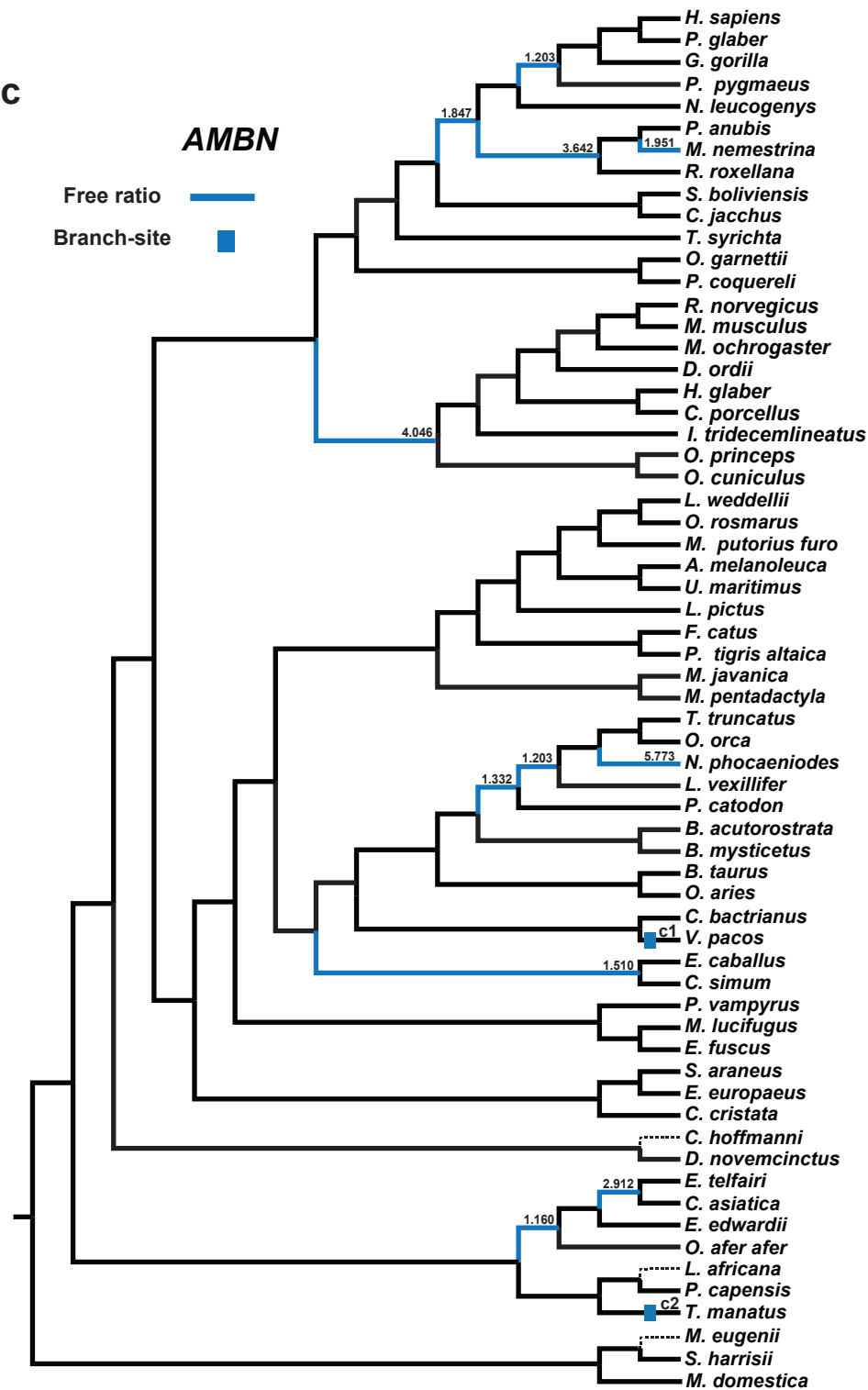

d

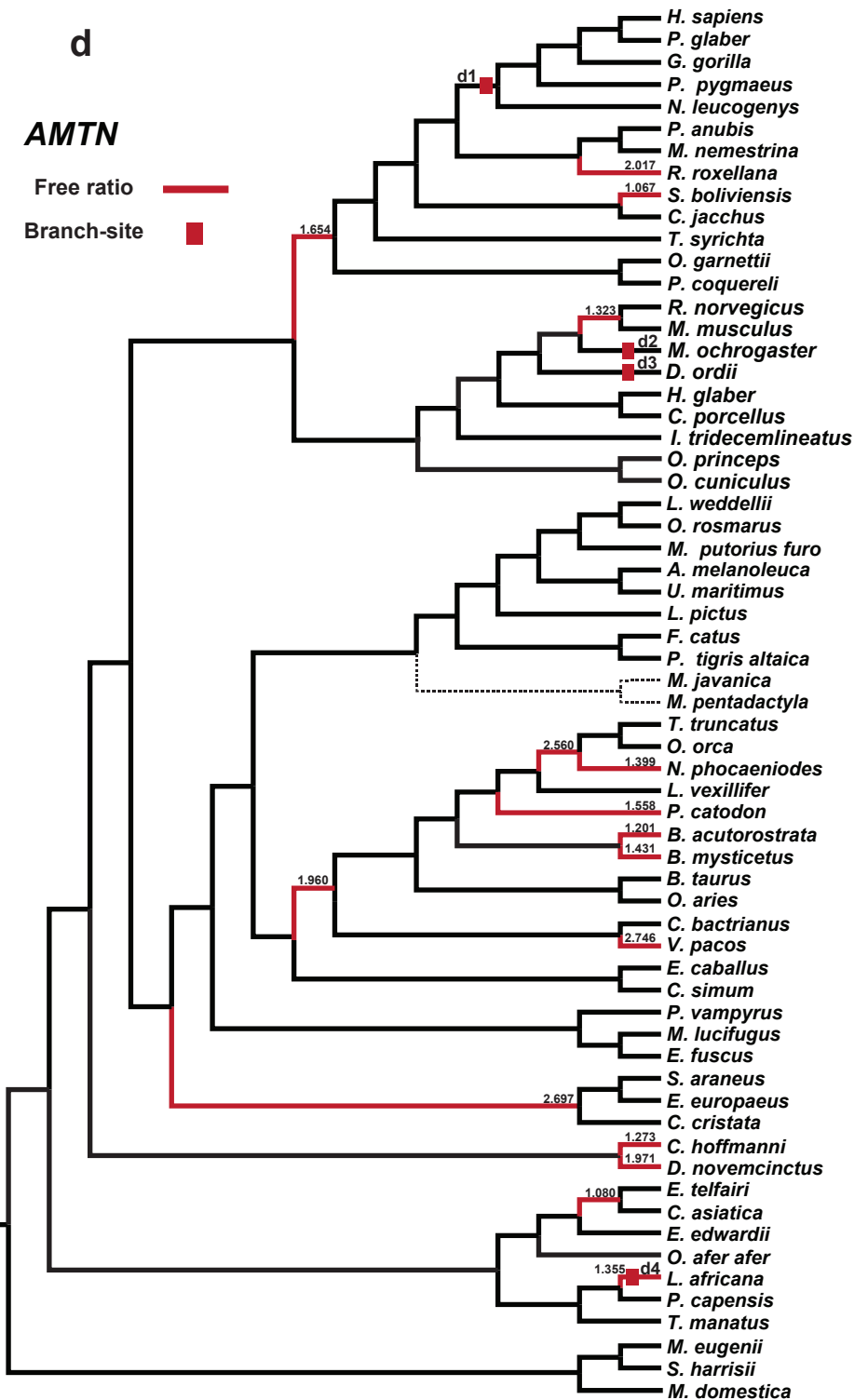

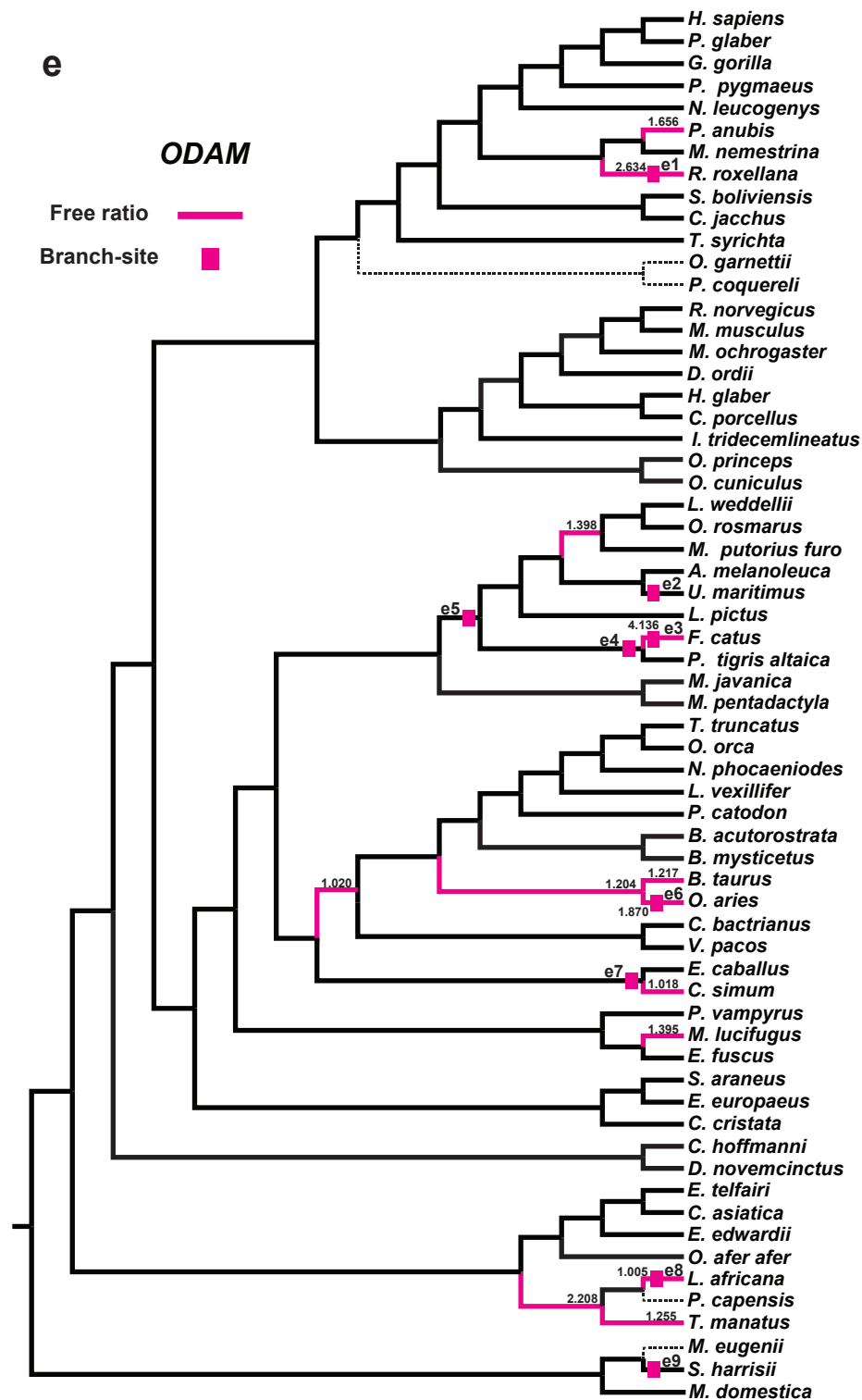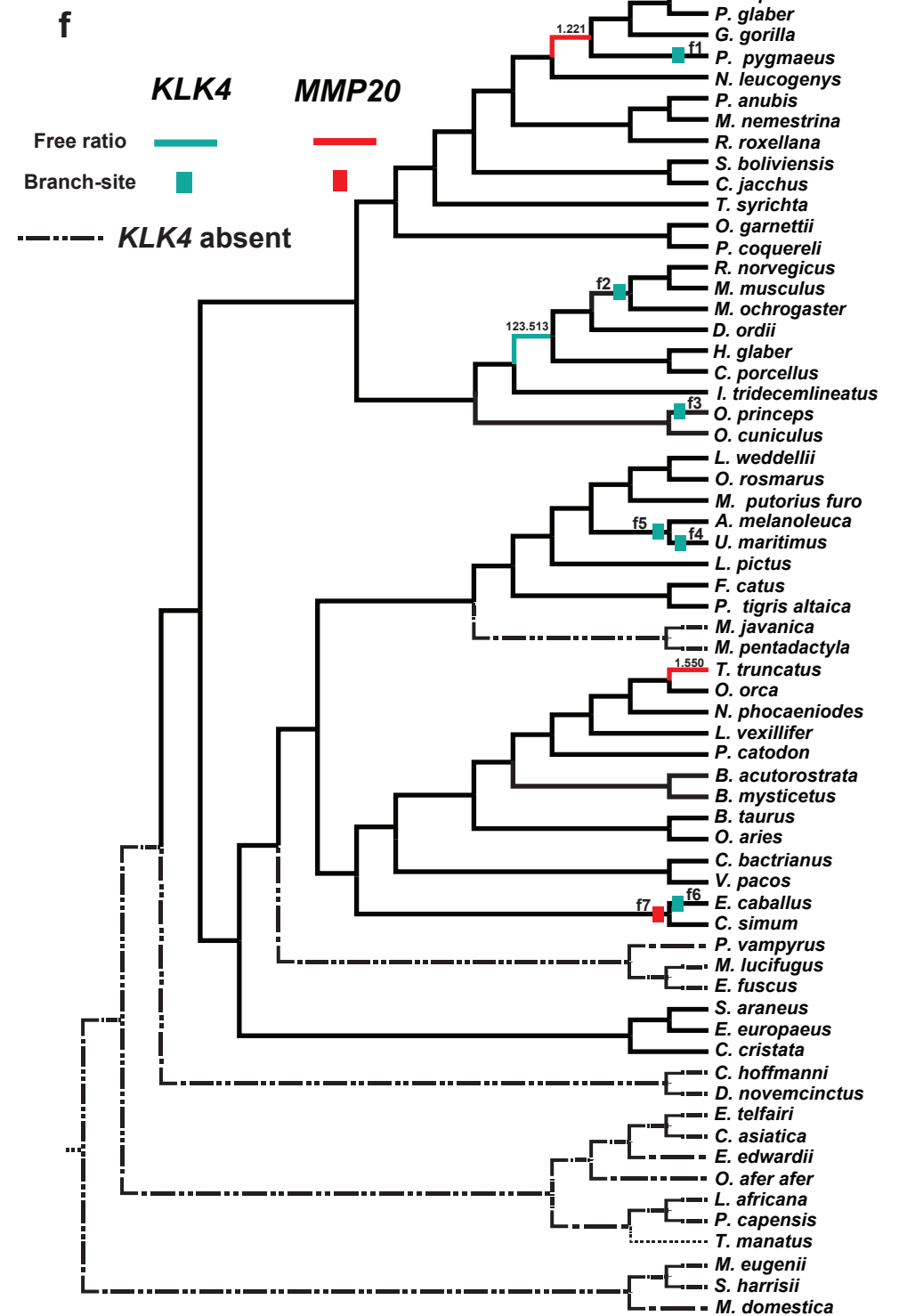

g

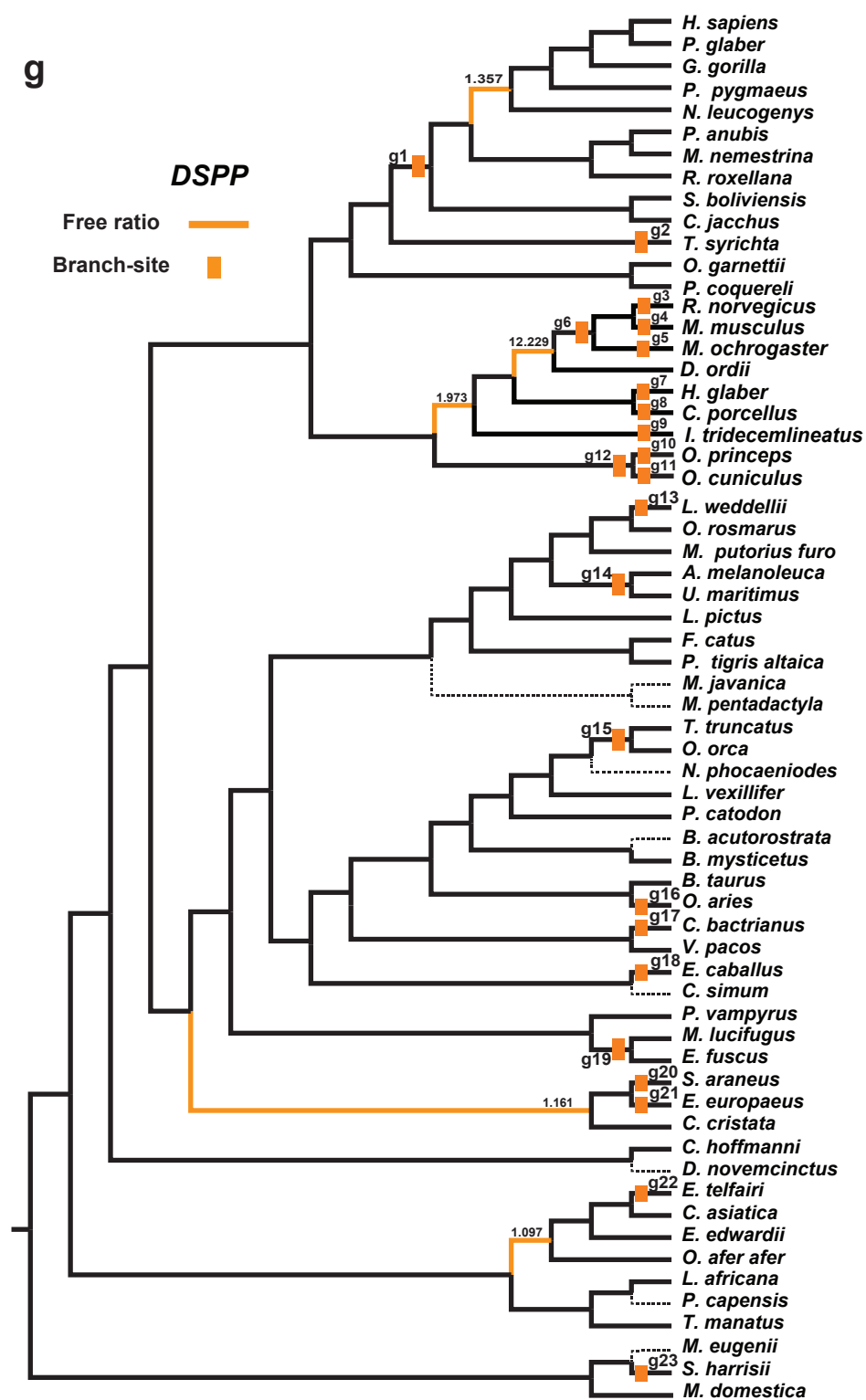

h

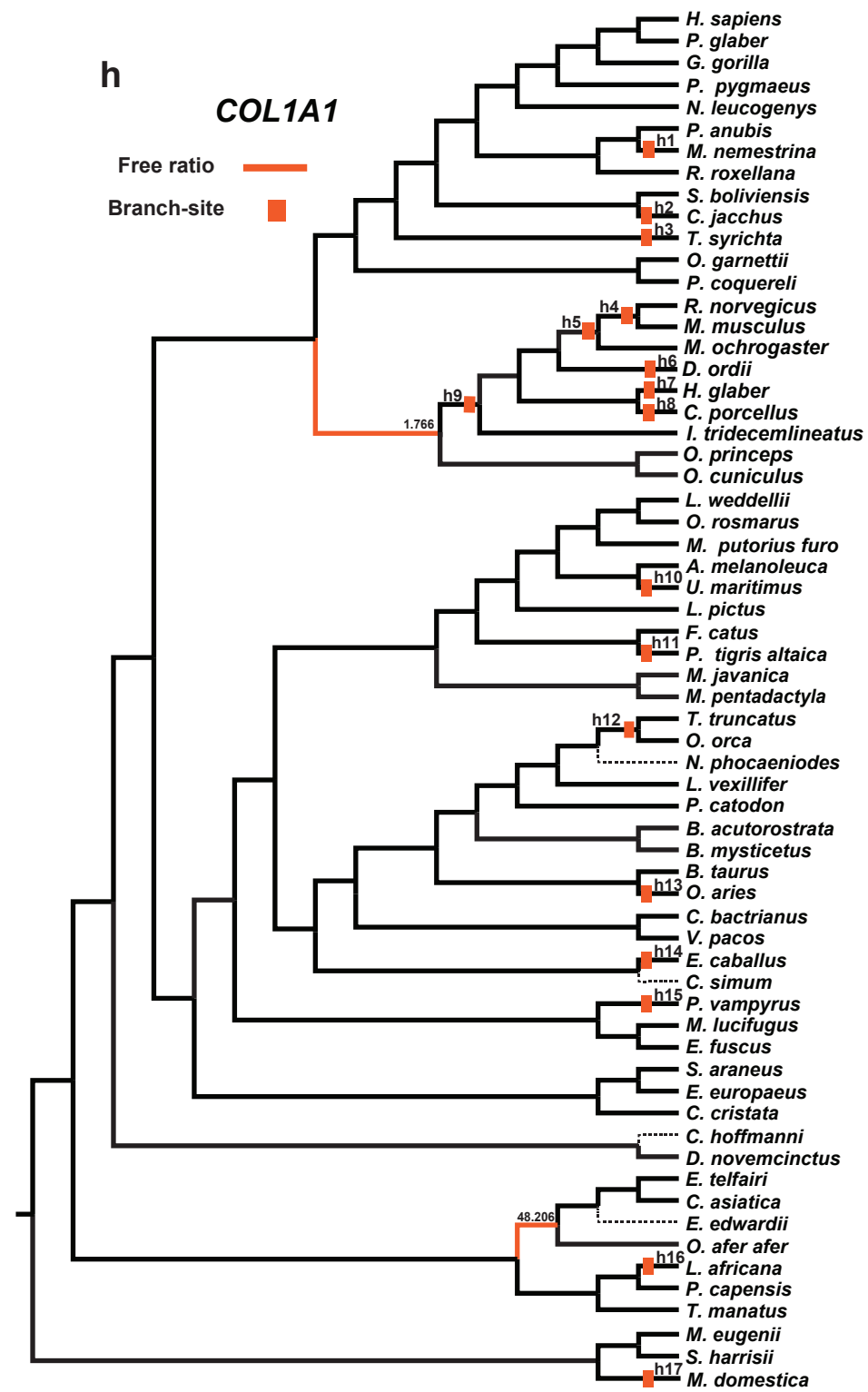

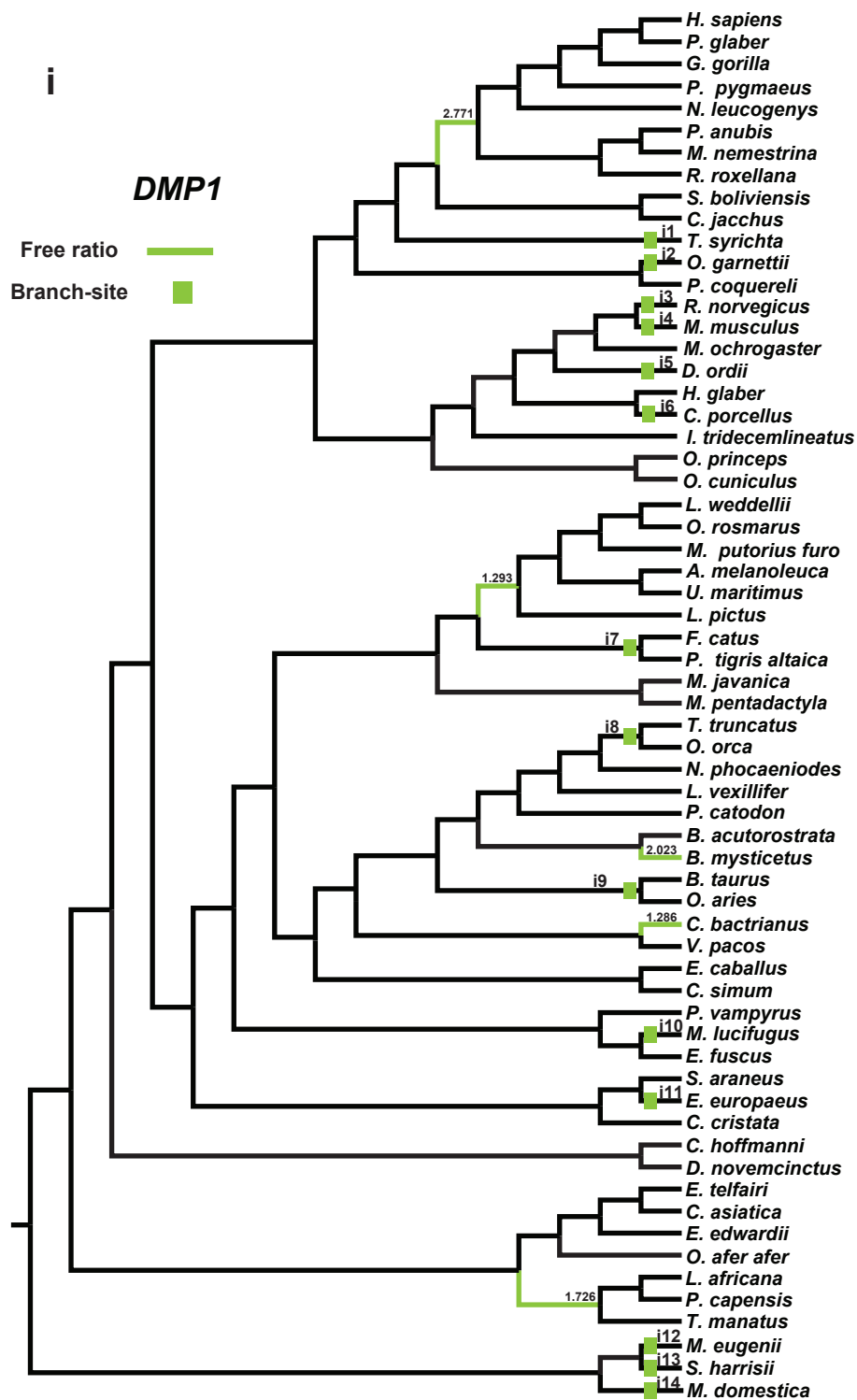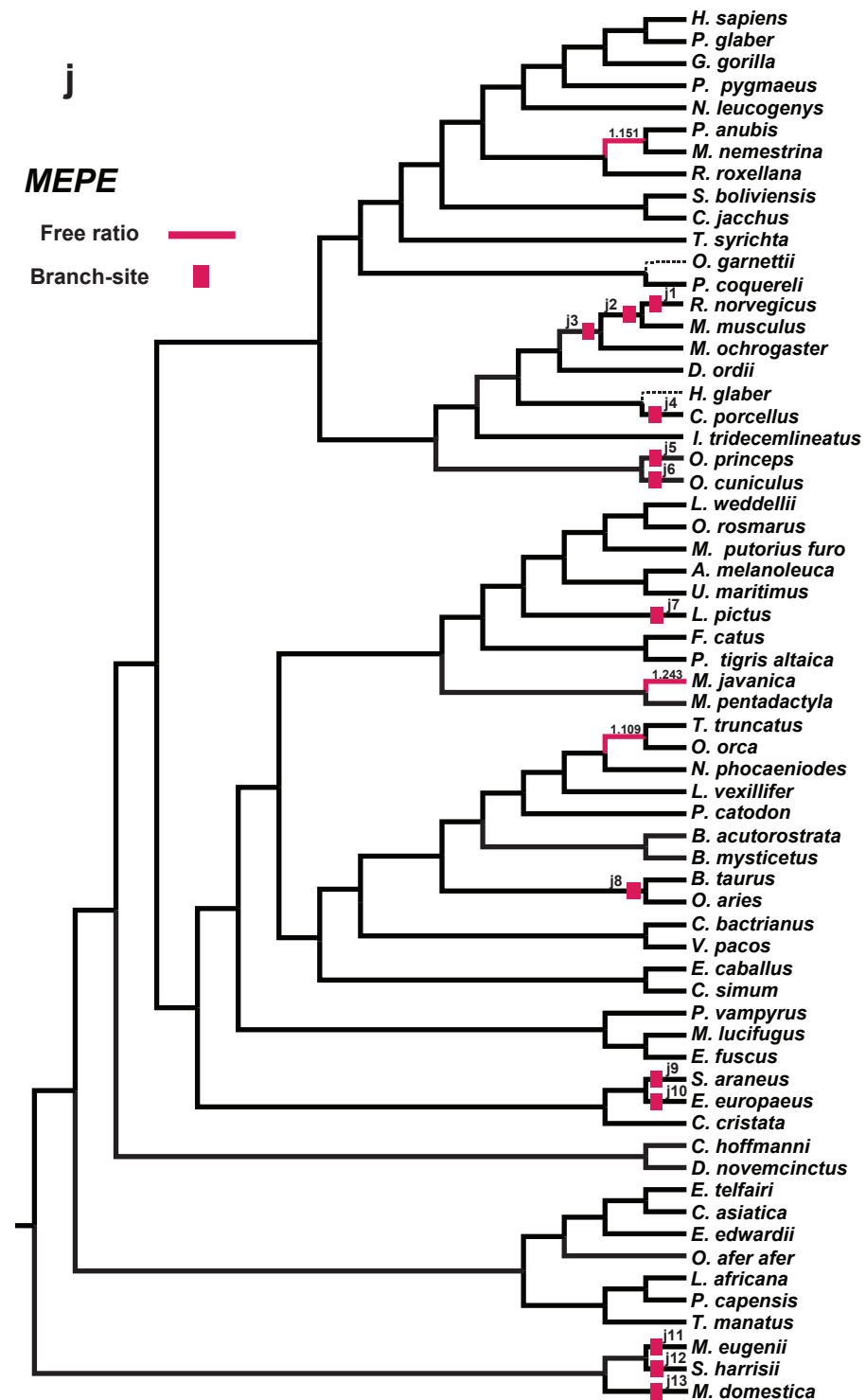

k

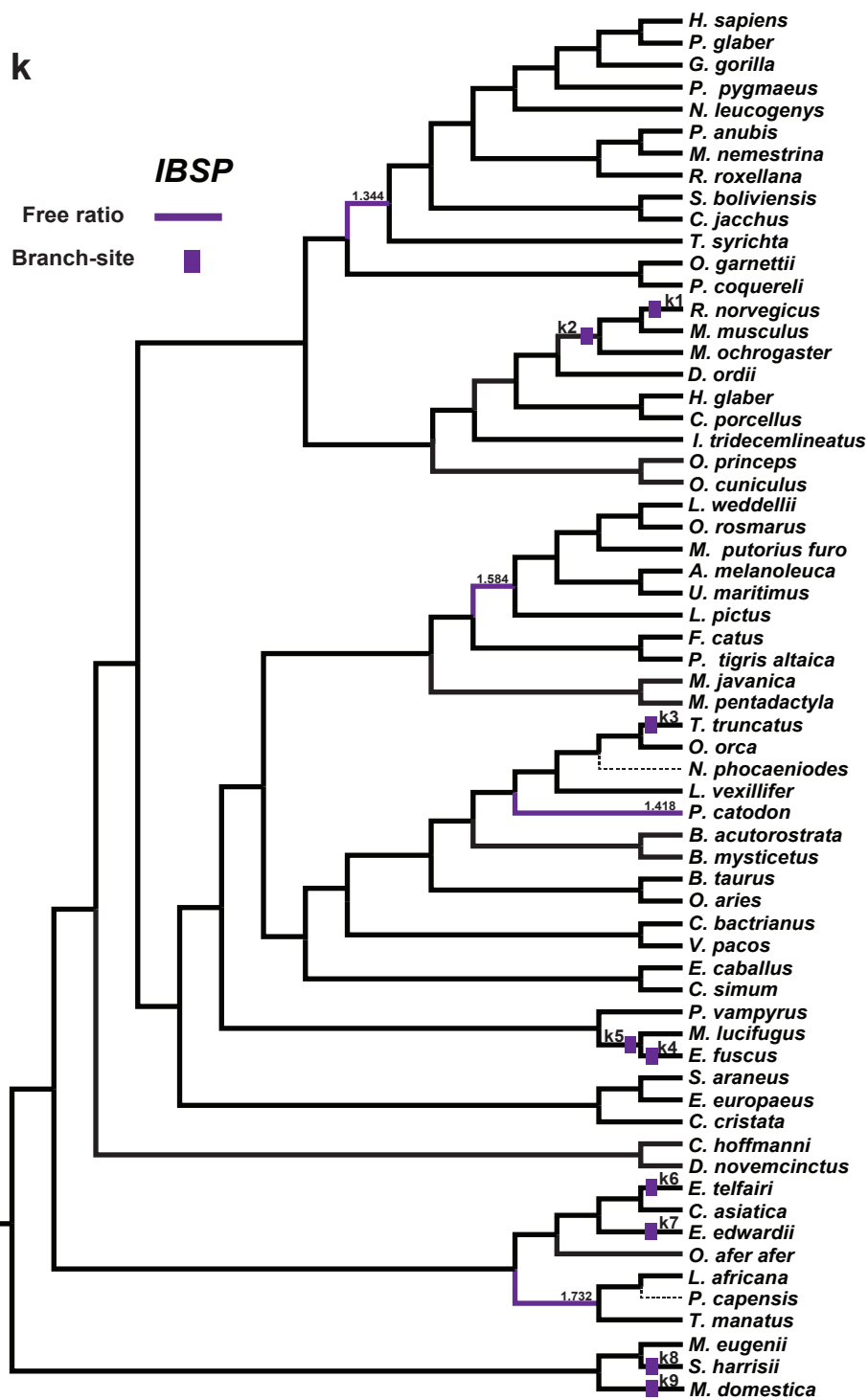

l

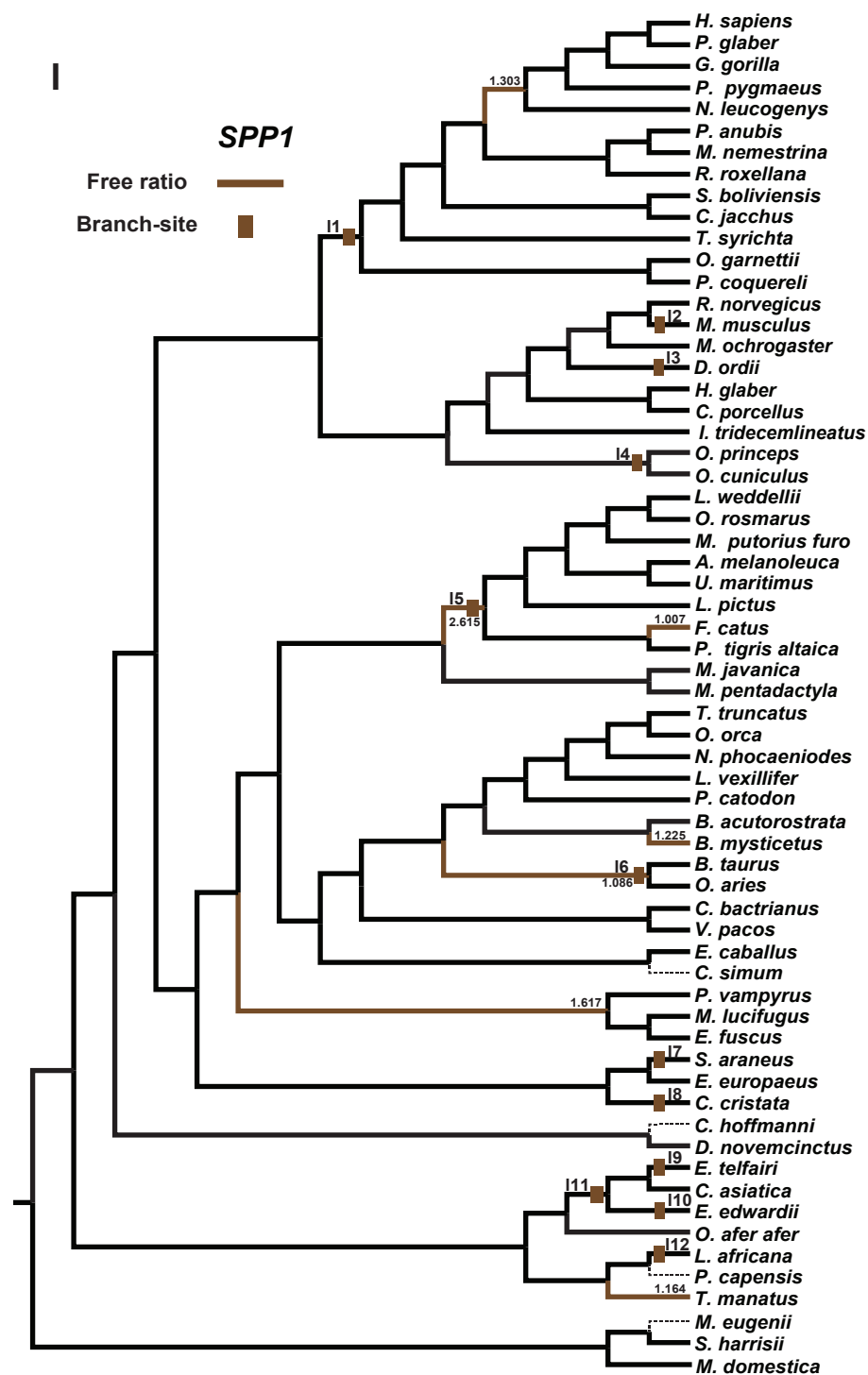

Supplement: Supplementary file 2 — Supplementary file2—Figure S1 Distribution of positive selection of each gene among three feeding mammals. Dashed line in species tree means there’s no corresponding species and data used in analyses (PDF 1563 kb) [file 239_2021_10017_MOESM2_ESM.pdf]

Enamel-related genes ■

Dentine-related genes ■

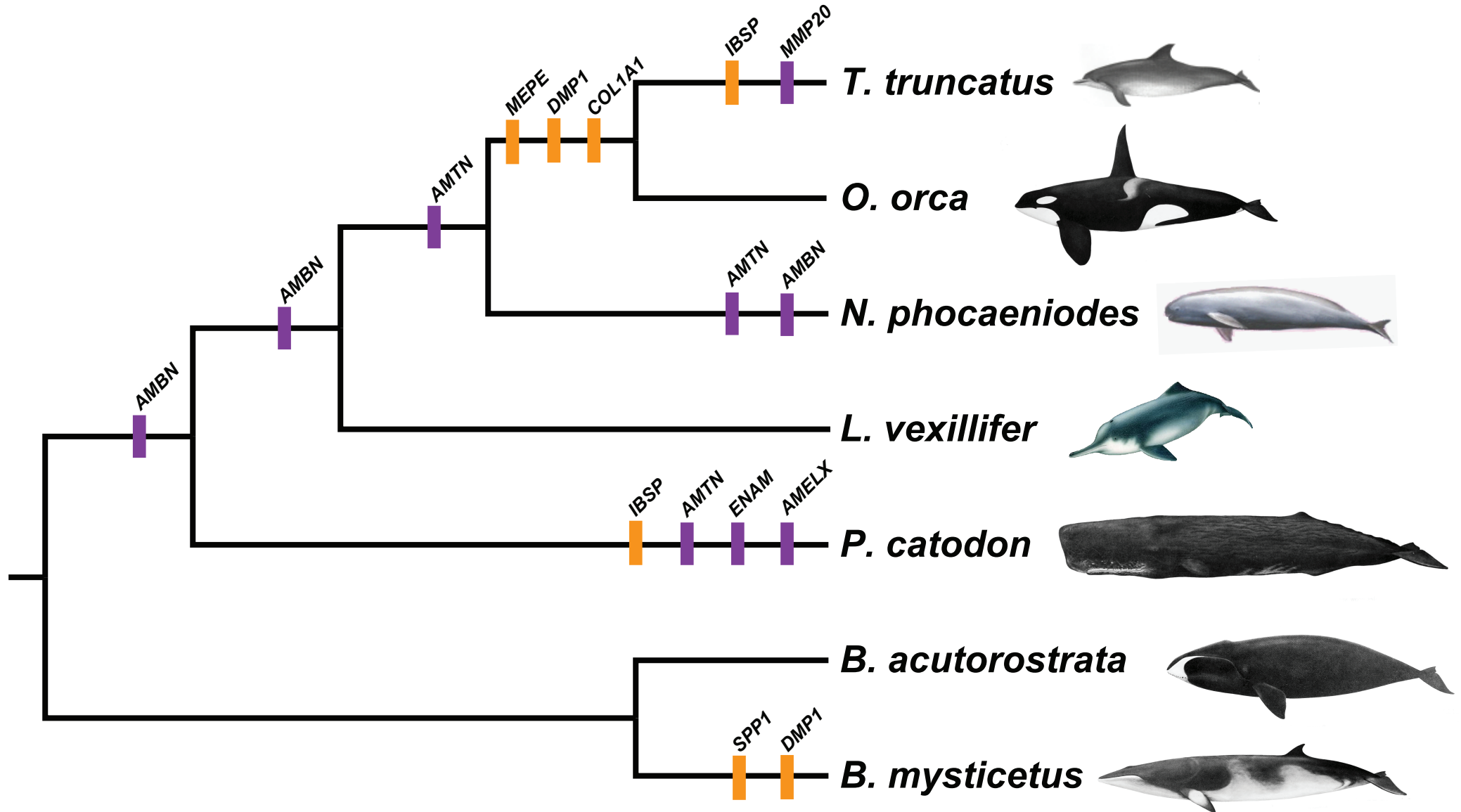

Supplement: Supplementary file 3 — Supplementary file3—Figure S2 Distribution of positive selection on cetacean phylogeny. (images are obtained from WiKi website: https://en.wikipedia.org/) (PDF 3933 kb) [file 239_2021_10017_MOESM3_ESM.pdf]

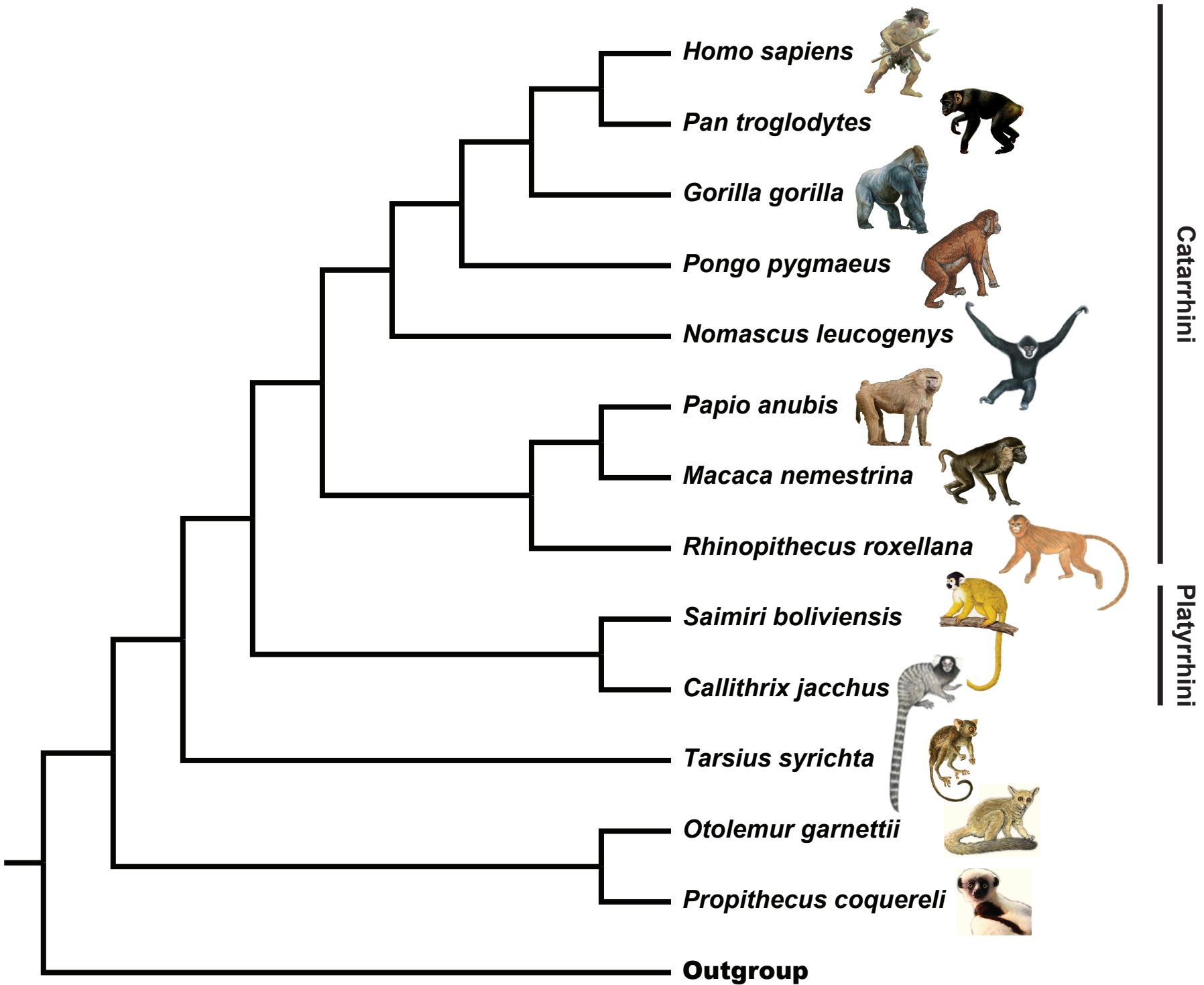

Haplorrhini

Strepsirrhini

Supplement: Supplementary file 4 — Supplementary file4—Figure S3 The primate phylogeny and dataset used for association analysis between root-to-tip ω and average enamel thickness.(Images are derived from WiKi website: https://en.wikipedia.org/wiki/Primate; AWD website: http://animaldiversity.org/, respectively) (PDF 6730 kb) [file 239_2021_10017_MOESM4_ESM.pdf]
