# Supplementary material for: Molecular Evolution of Tooth-Related Genes Provides New Insights into Dietary Adaptations of Mammals
Source: J Mol Evol. 2021 Jul 21;89(7):458–71. doi: 10.1007/s00239-021-10017-1 (PMC8318974; doi:10.1007/s00239-021-10017-1)

a. ***AMELX*** **Human sequence as the reference sequence**

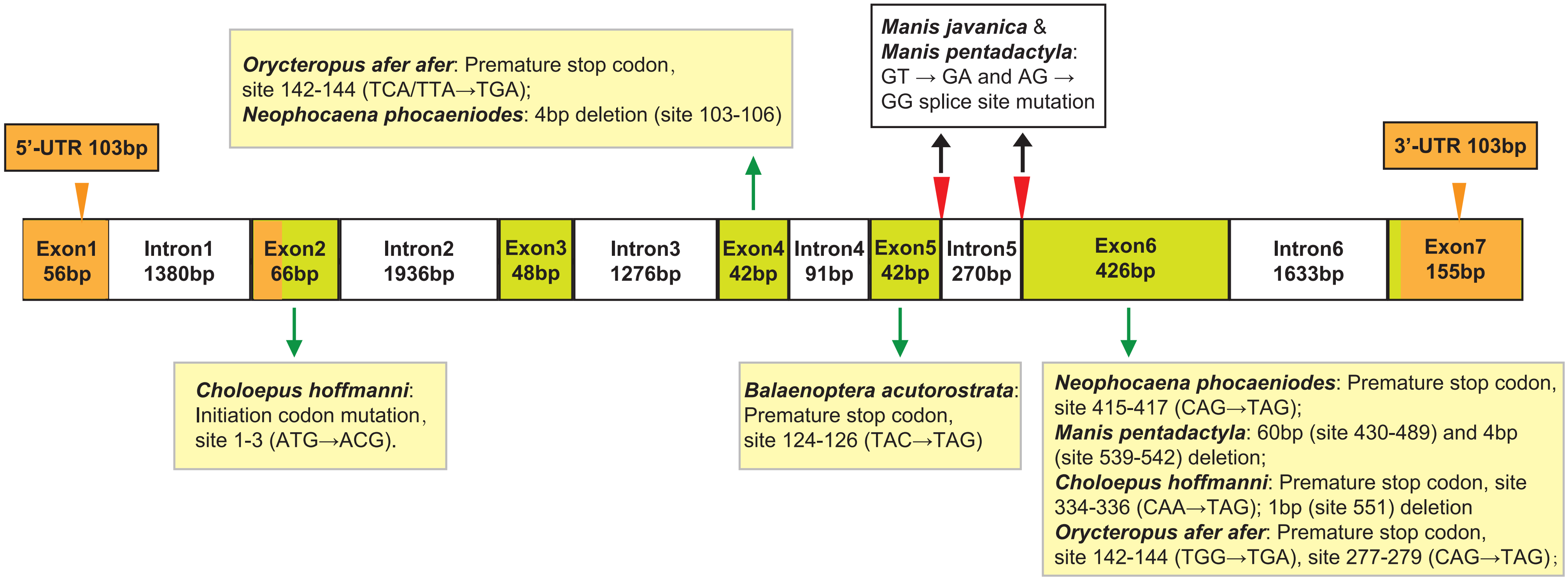

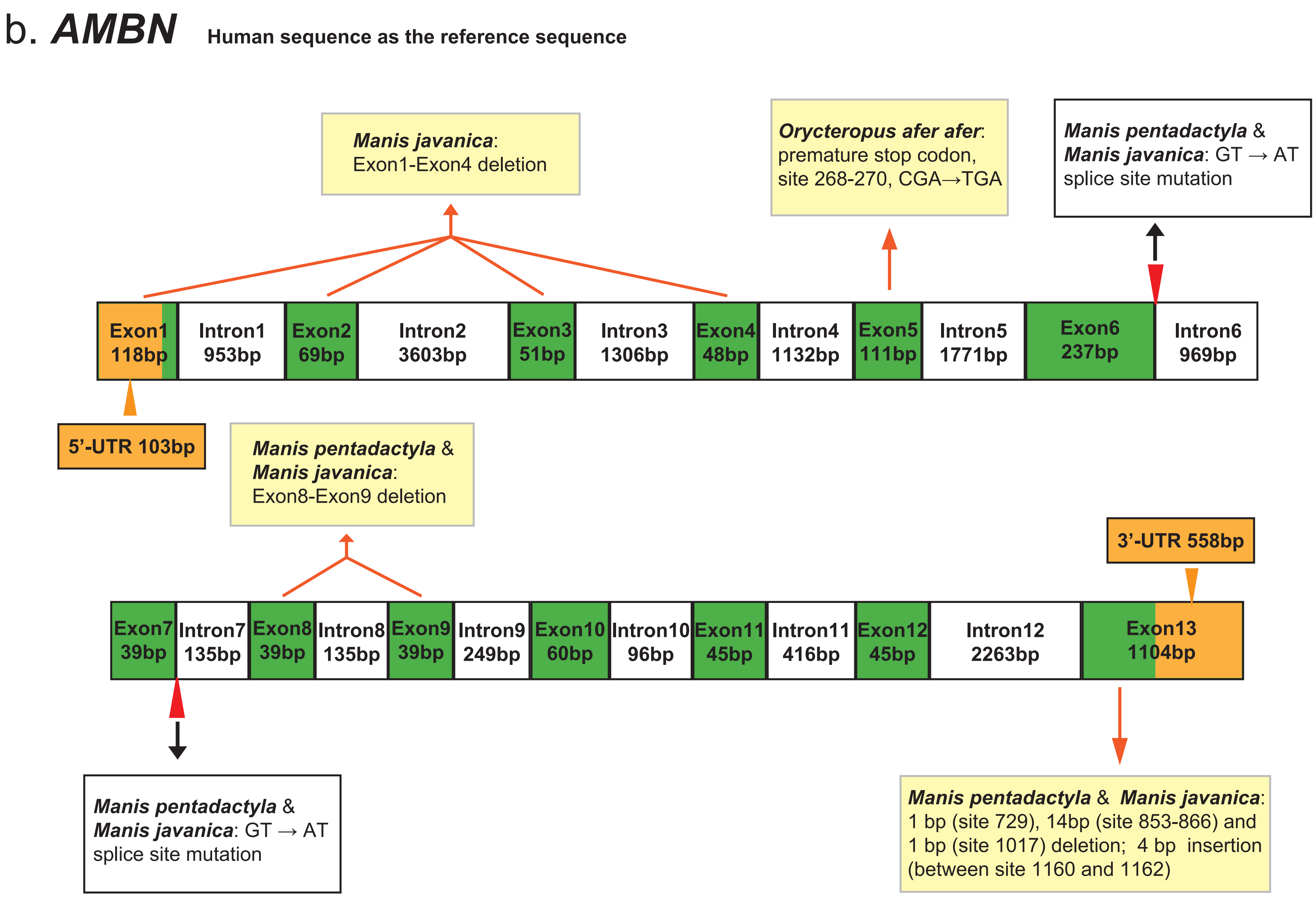
b. AMBN
Human sequence as the reference sequence
Manis javanica: Exon1-Exon4 deletion
Orycteropus afer afer: premature stop codon, site 268-270, CGA→TGA
Manis pentadactyla & Manis javanica: GT → AT splice site mutation
Exon1 118bp
Intron1 953bp
Exon2 69bp
Intron2 3603bp
Exon3 51bp
Intron3 1306bp
Exon4 48bp
Intron4 1132bp
Exon5 111bp
Intron5 1771bp
Exon6 237bp
Intron6 969bp
5'-UTR 103bp
Manis pentadactyla & Manis javanica: Exon8-Exon9 deletion
3'-UTR 558bp
Exon7 39bp
Intron7 135bp
Exon8 39bp
Intron8 135bp
Exon9 39bp
Intron9 249bp
Exon10 60bp
Intron10 96bp
Exon11 45bp
Intron11 416bp
Exon12 45bp
Intron12 2263bp
Exon13 1104bp
Manis pentadactyla & Manis javanica: GT → AT splice site mutation
Manis pentadactyla & Manis javanica: 1 bp (site 729), 14bp (site 853-866) and 1 bp (site 1017) deletion; 4 bp insertion (between site 1160 and 1162)

c. *ENAM* Human sequence as the reference sequence

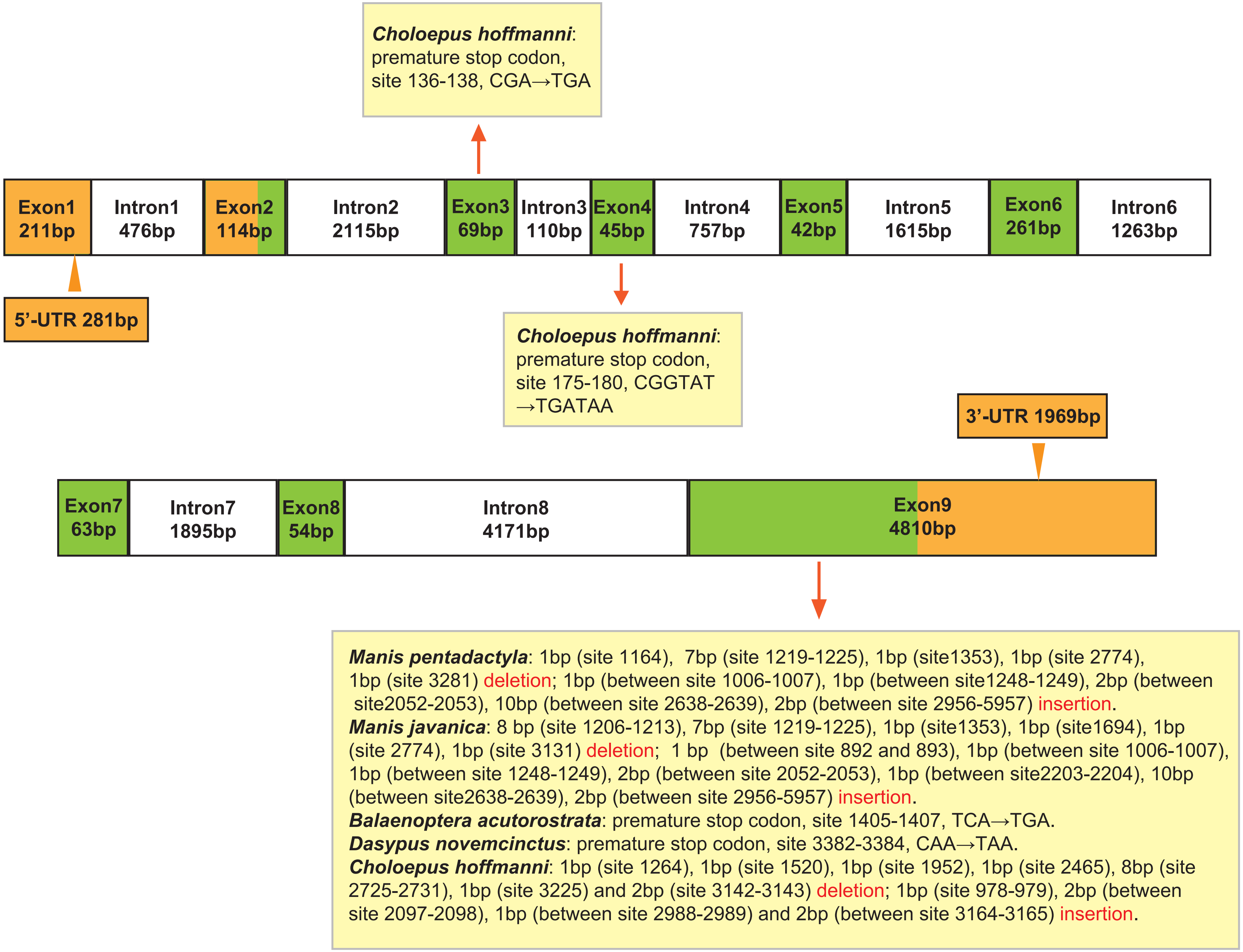

d. ***AMTN*** **Human sequence as the reference sequence**

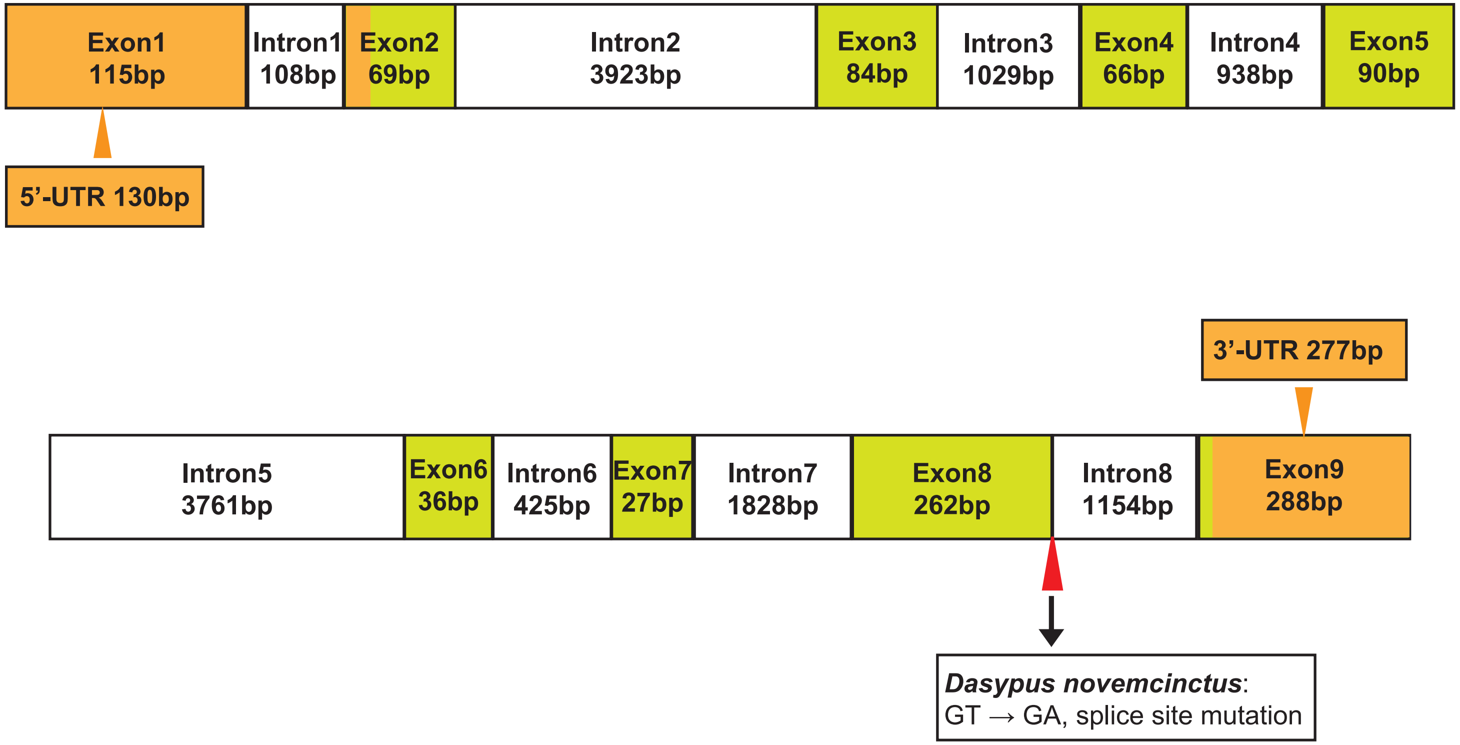

e. ***MMP20*** **Human sequence as the reference sequence**

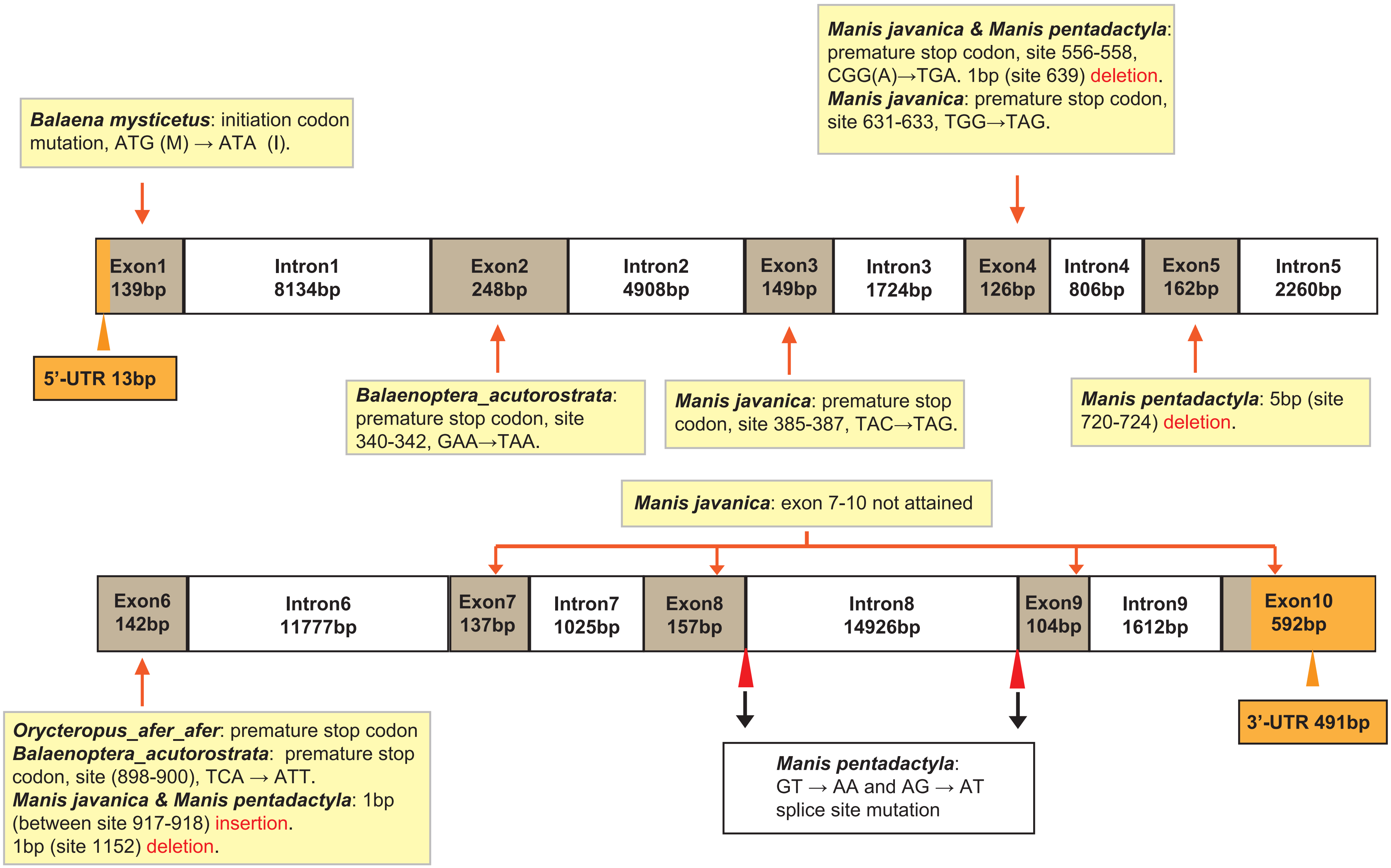

f. *ODAM* **Human sequence as the reference sequence**

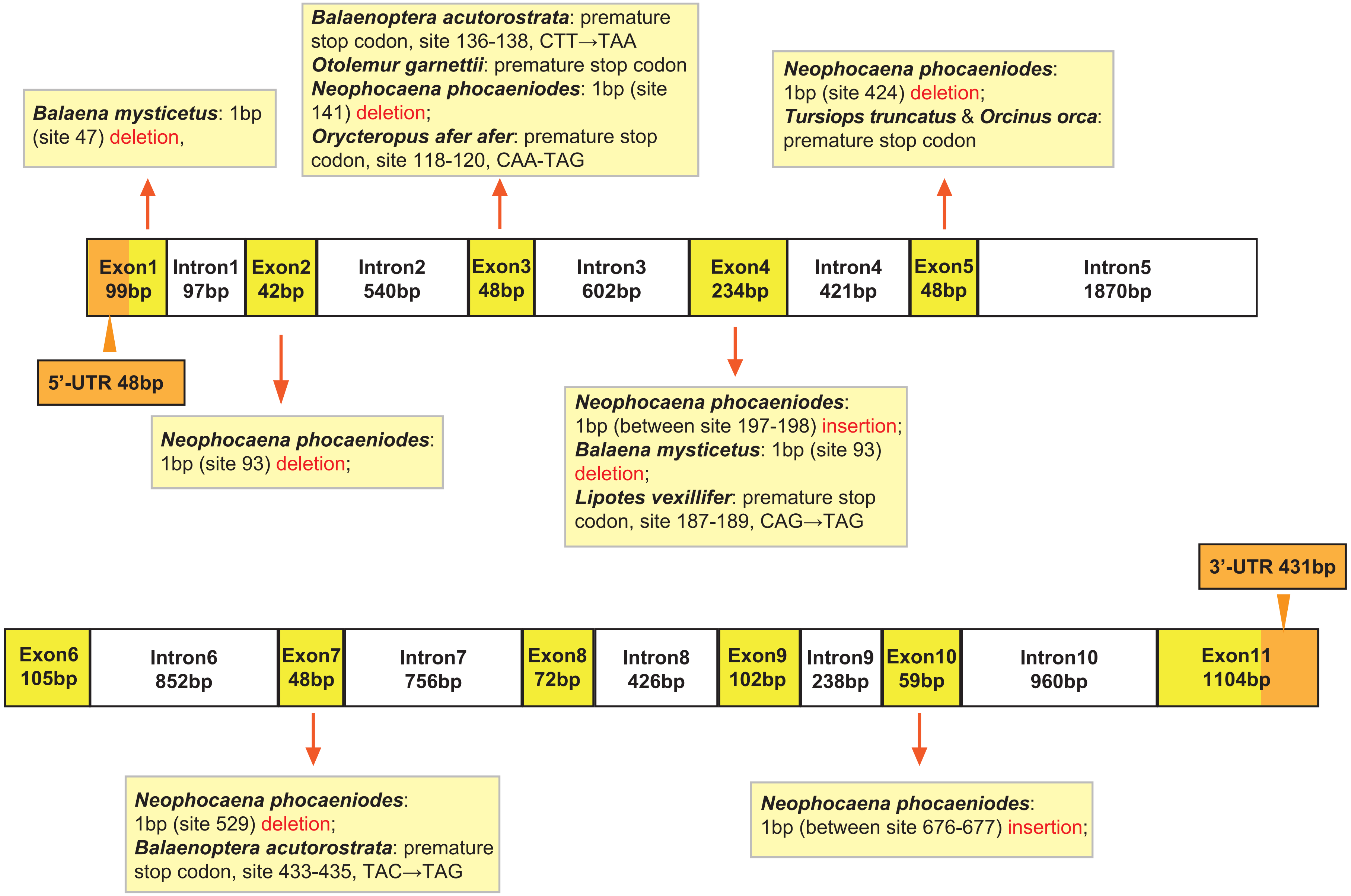

g. ***DSPP*** **Human sequence as the reference sequence**

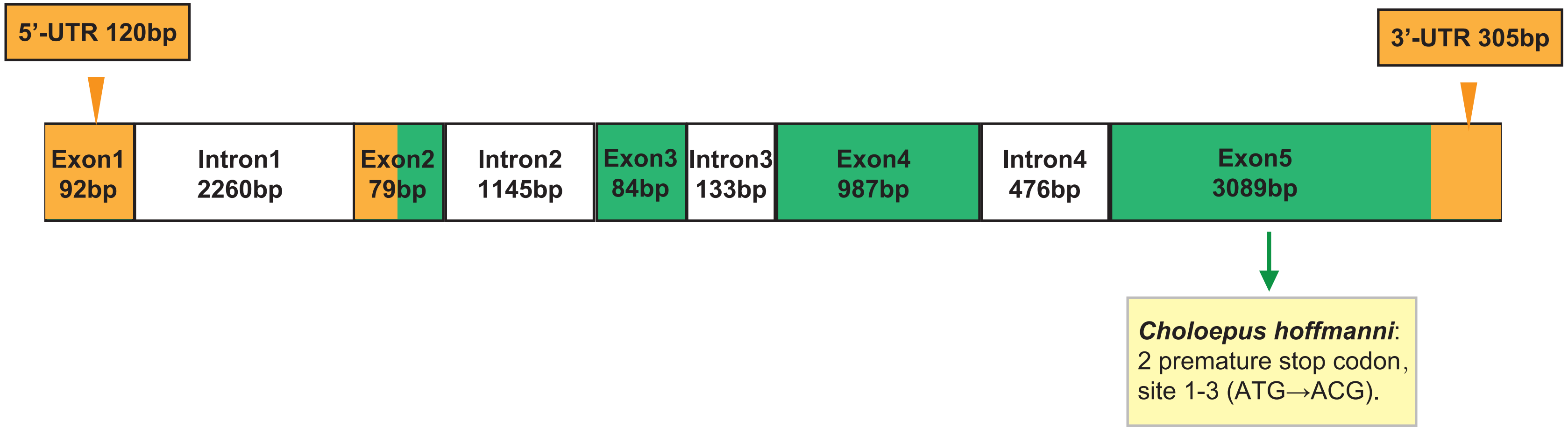

Supplement: Supplementary file 5 — Supplementary file5—Figure S4 The overview about the information of inactive mutation in some tooth-related genes (PDF 1242 kb) [file 239_2021_10017_MOESM5_ESM.pdf]
